# Investigating the Persuasive Effects of Testimonials on the Acceptance of Digital Stress Management Trainings Among University Students and Underlying Mechanisms: A Randomized Controlled Trial

**DOI:** 10.3389/fpsyg.2021.738950

**Published:** 2021-10-13

**Authors:** Jennifer Apolinário-Hagen, Lara Fritsche, Jeannette Wopperer, Frank Wals, Mathias Harrer, Dirk Lehr, David D. Ebert, Christel Salewski

**Affiliations:** ^1^Institute of Occupational, Social and Environmental Medicine, Centre of Health and Society, Faculty of Medicine, Heinrich Heine University Düsseldorf, Düsseldorf, Germany; ^2^Department of Health Psychology, Faculty of Psychology, University of Hagen, Hagen, Germany; ^3^Faculty of Psychology, University of Hagen, Hagen, Germany; ^4^Department of Clinical Psychology and Psychotherapy, Friedrich-Alexander-University Erlangen-Nuremberg, Erlangen, Germany; ^5^Department of Health Psychology and Applied Biological Psychology, Institute of Psychology, Leuphana University Lueneburg, Lueneburg, Germany; ^6^Department of Sport and Health Sciences, Technical University of Munich, Munich, Germany

**Keywords:** eHealth, mental health, stress, attitude, intention, students, personal narratives

## Abstract

**Objective:** This experiment aims to investigate the influence of narrative information varying in the degree of perceived similarity and source credibility in supplemented testimonials on the acceptance of digital mental health services (digi-MHSs).

**Methods:** In fall 2020, *n*=231 university students were randomly assigned to an active control group (aCG, *n*=55, “information only”) or one of three intervention groups (IGs) receiving information plus different testimonials being presented either by nonacademic staff (IG1, *n*=60), university students (IG2, *n*=58) or experts (IG3, *n*=58). We assessed mediation effects of similarity and credibility on acceptance in terms of attitudes and usage intentions.

**Results:** Exposure to testimonials was associated with higher usage intentions (*d*=0.50) and more positive attitudes toward digi-MHSs (*d*=0.32) compared to mere information (aCG). Regarding source-related effects, one-way ANOVA showed group differences in intentions ( $\eta_{p}^{2}$ =0.13) that were significantly higher after exposure to testimonials targeted at students than in the other groups after adjusting for baseline intentions ( $\eta_{p}^{2}$ =0.24). Concerning underlying mechanisms, there were full mediation effects of similarity (IG1 versus IG2) on attitudes [95%CI (0.030, 0.441)] and intentions to use digi-MHSs [95%CI (0.100, 0.528)] and of credibility on attitudes [IG2 versus IG3; 95%CI (−0.217, −0.004)], all favoring students’ testimonials.

**Conclusion:** Overall, this study indicates that the acceptance of digi-MHSs can be substantially increased by providing a simple, context-sensitive information intervention, including testimonials by university students. Since we identified mediating effects of credibility on cognitive attitudes and similarity on affect-driven intentions, a future trial could vary these features using narrative versus statistic information on digi-MHSs.

## Introduction

Mental health promotion for college and university students has become a central topic on the international research and health policy agenda in recent years, given the increasing prevalence for psychological problems in this population (Cuijpers et al., 2019). Still, there is an immense discrepancy between the supposed need and actual uptake of mental health services by students worldwide (Auerbach et al., 2018). Moreover, since the onset of the Covid-19 pandemic, university students have been found to experience further psychosocial strain and help-seeking barriers (Benjet, 2020; Davenport et al., 2020; Kohls et al., 2021). Digital mental health services (digi-MHSs) provide additional options to increase the availability of health promotion and treatment offers (van Daele et al., 2020). In general, digi-MHSs include a broad range of interventions differing in theory base [e.g., internet-delivered cognitive behavioral therapy (iCBT)], application fields (e.g., stepped care), guidance (e.g., asynchronous feedback), and technical implementation (e.g., virtual reality; Ebert et al., 2019). To date, solid evidence exists for the efficacy of digi-MHSs for improving subjective wellbeing or coping with stress, anxiety, and depression across student populations (Harrer et al., 2018; Lattie et al., 2019). As an example, online stress management trainings have been demonstrated to be efficacious for distressed to moderately depressed traditional and nontraditional university students facing multiple challenges, like study-work-family-conflicts (Harrer et al., 2021).

Interestingly, research indicated a higher acceptance of digi-MHSs among university students with personal use experience, but the utilization rates of existing digital interventions remain very low (Dunbar et al., 2018; Hadler et al., 2021; Lavergne and Kennedy, 2021). Potentially, suitable digi-MHSs are yet not well known and thus seldom used by university students despite overall positive attitudes (Mayer et al., 2019; Apolinário-Hagen et al., 2021). Although many university students appear ready to use digital health solutions, they still report difficulties in finding reliable information online (Machleid et al., 2020; Dadaczynski et al., 2021). Accordingly, the willingness to use digital media for mental health purposes depends on appropriate, easy accessible information regarding core requirements, like data security (Montagni et al., 2020). Uncertainties grounded on limited or conflicting information, besides unmet preferences, may thus impede the adoption of evidence-based psychological services (Cunningham et al., 2014, 2017).

Recent research suggests that tailored fact-based psychoeducational information can help increase intentions to use mental health services among university students (Ebert et al., 2018). Under “real world” conditions, consumer choices are oftentimes based on the opinions, anecdotes, or recommendations from trustworthy sources. Hence, a commonly applied practice is to make use of the supposed impact of user reviews, including star ratings, quality claims, and expert statements, especially in order to advertise commercial mental health apps (Apolinário-Hagen et al., 2018a; Larsen et al., 2019). Narrative messages can facilitate experience-based heuristic decisions, based on rules of thumb or practical examples. Simple heuristics are particularly useful in pragmatic decisions in new situations in daily life (e.g., reducing complexity, dealing with limited information; Gigerenzer and Gaissmaier, 2011).

Consequently, dual-processing models, like the *Elaboration Likelihood Model* (Petty et al., 2009) and the *Heuristic-Systematic Model* (Chaiken, 1980), propose two main pathways of persuasion or attitude change (analytical versus heuristic) that depend on the individual ability and motivation to process health messages as well as various contextual factors. To date, though, knowledge on the specific influence of different features of mental health information, especially of those being related to the context (e.g., expert heuristics, reputation) instead of the content (e.g., facts like duration, themes), is limited and inconclusive. Most research on health-related testimonials has dealt with prevention and treatment choices regarding somatic disorders and yielded mixed findings on the benefits of statistical over narrative information, like testimonials (e.g., Zebregs et al., 2015; Perrier and Martin Ginis, 2017). Among message recipients without own experience with mental health interventions, testimonials by past users may be more influential on hypothetical treatment choices than among recipients with first-hand treatment experience (Pruitt et al., 2012). In addition, it may be possible that educational material combining fact-based statistical information with testimonials can improve attitudes toward digi-MHSs such as iCBT among both concerned and unconcerned people (Soucy et al., 2016).

Regarding variables related to attitude change, perceived similarity between testimonial sources and oneself as well as source credibility have been identified as persuasive factors across various health communication fields (Green and Clark, 2013; Shen et al., 2015; Shaffer et al., 2018). Medical students, for instance, have been shown to prefer digital interventions that are tailored to students and approved by trustworthy academic sources (Dederichs et al., 2021). Accordingly, testimonials on digi-MHSs may represent a simple way to facilitate their acceptance among university students as they are seldom familiar with such offers and may thus likely be affected by heuristics based on perceived similarity or source credibility (Quintero Johnson et al., 2017, 2021).

Taken together, little is known about the usefulness of testimonials as a widely applied marketing tool to promote the acceptance of digi-MHSs among university students as well as mechanisms underlying testimonial effects, which could help tailor health messages.

### Objectives

This study aimed to investigate the influence of information varying in the degree of supposed similarity of narrators with oneself and source credibility of testimonials compared to mere information on the acceptance of digi-MHSs (in terms of attitudes and intentions) among university students. Another purpose was to explore whether perceived similarity and source credibility mediate the influence of testimonials on the acceptance of digi-MHSs, like digital stress management trainings. In view of the inconclusive evidence of testimonials effects, we postulate three research questions (RQs).

**RQ1:** *Is there an added value of testimonials as a supplement to neutral information compared to mere information regarding the acceptance of digi-MHSs among university students?*

We assumed positive influences on (RQ1a) attitudes and (RQ1b) intentions to use digi-MHSs among university students after the exposure to information augmented with testimonials compared to information only.

**RQ2:** *Are there differences in students’ acceptance of digi-MHSs following information varying in source credibility and perceived similarity?*

We explored differences in (RQ2a) attitudes and (RQ2b) intentions based on the exposure to testimonials from different sources (i.e., employees working outside of academia versus university students versus qualified academic experts). We supposed a higher influence of university students’ and experts’ testimonials compared to nonacademic staff testimonials and information only.

**RQ3:** *Do perceived similarity and source credibility mediate the effects of different testimonial sources on students’ acceptance of digi-MHSs?*

Concerning mechanisms underlying testimonial effects, we explored mediation effects of (RQ3a) perceived similarity with oneself and (RQ3b) source credibility on students’ attitudes and intentions.

## Materials and Methods

### Study Design and Interventions

**Figure 1:**
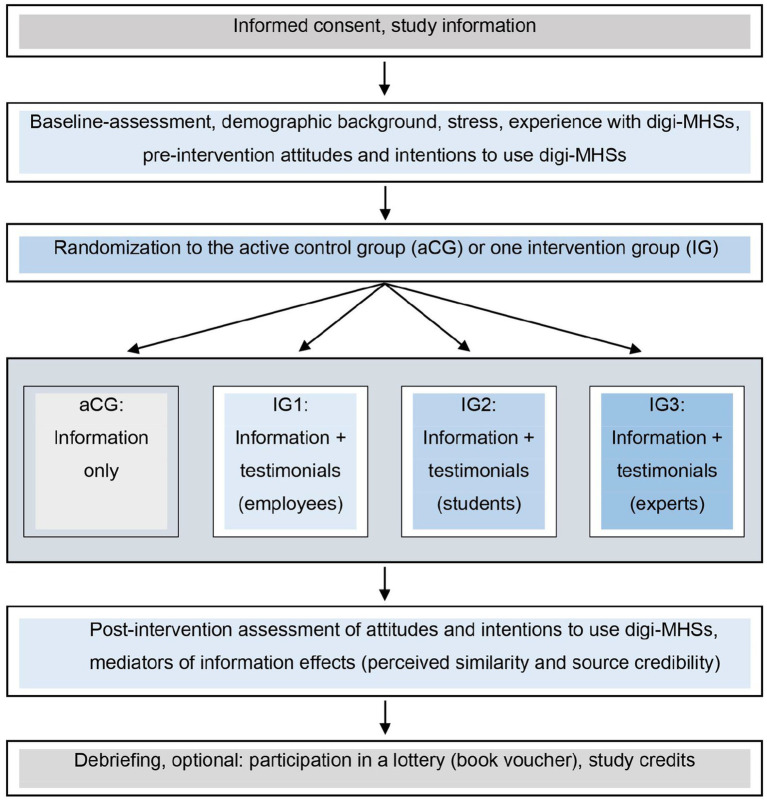
Study flow chart. Procedure of the online experiment comparing attitudes and intentions to use digital mental health interventions (digi-MHSs) between three narrative intervention groups (IGs) receiving information plus various testimonials and the active control group (aCG) receiving information only.

In a randomized controlled trial with four parallel information groups (study arms), we assessed differentiated effects of brief, written testimonials in addition to text-based information on attitudes and intentions to use digi-MHSs, as shown in Figure 1.

The anonymously conducted survey-based online experiment was designed based on previous work (Apolinário-Hagen et al., 2018a, 2021). Using a computer-based algorithm (balanced, 1:1:1:1) implemented in the online survey tool *Unipark* (Questback), participants were randomly assigned to an active control condition (aCG=“information only”) or to one of three narrative intervention groups (IGs). The IGs involved information differing in supplemented fictitious testimonials that were either presented by staff outside of academia (IG1), university students (IG2), or academic experts (IG3) in order to vary similarity to students (IG2 versus IG1 and IG3) and credibility (IG3 versus IG2 and IG1). All participants received the same general information on digi-MHSs, while the IGs additionally received three text-based testimonials that were constructed in orientation to real-world examples, theoretical considerations and pretested stimulus material. The complete contents of the interventions are shown in Supplementary Material 1. In contrast to the pilot studies (e.g., Apolinário-Hagen et al., 2021), we did not include brands of existing digi-MHSs, albeit the described service was based on an evidence-based, guided digital stress management training (Ebert et al., 2016; Harrer et al., 2021). Moreover, the revised study material was designed more consistently and less detailed (e.g., exclusively scoping on stress prevention, missing age of testimonial sources) while trying to achieve external validity (e.g., emulating existing testimonials, experts as additional source).

The valence of testimonials was positive, focused on advantages (personal experience in IG1 and IG2, third-party: IG3), and the intended effect was persuasion (c.f., Shaffer and Zikmund-Fisher, 2013). All testimonials were fictional in order to control for contextual factors related to varying knowledge and popularity of experts. The online survey was pretested with *n*=10 university students. The average completion time was between 12 and 17min. This study was approved by the ethic committee of the University of Hagen, Faculty of Psychology, Germany (EA_278_2020).

### Sample and Recruitment

Inclusion criteria were self-reported student status, age of at least 18years, and provided consent (click-to-agree). Data were collected online between September 3, 2020 and October 3, 2020 using *Unipark*. German-speaking participants were recruited using the *virtual lab* and *Moodle* groups of the University of Hagen as Germany’s only state distance-learning university, social media (e.g., *Facebook*), flyers with QR code distributed across different German universities and emails (e.g., student representatives). Psychology undergraduate students could receive study credits, while all completers had the chance to win book vouchers. Regarding the required sample size, *a priori* power analyses using G*Power (Faul et al., 2007) indicated *n*=170 for RQ1 [two-tailed independent-sample *t*-test, unequal group ratio 3:1, moderate effect size (*d*=0.5), power=0.8] and *n*=180 for RQ2 [one-way ANOVA, power=0.80, alpha=0.05, moderate effect (*f*^2^=0.0.25)], respectively.

### Measures and Procedure

The survey consisted of three main parts: (1) baseline, (2) intervention, and (3) post-intervention assessment. Different validated and pre-tested self-constructed scales were used for assessing information effects on the acceptance of digi-MHSs, as illustrated in Table 1.

**Table 1:** Constructs and psychometric data on the measured variables.

Construct/scale	Items	Min.	Max.	*Mean*	*SD*	Cronbach’s *α*
***Baseline/pre-intervention***						
Intentions (UTAUT, pre)^a^	3	1.33	7.00	4.53	1.01	0.67
Attitude (PU, pre)^a^	3	2.00	7.00	5.23	0.91	0.85
Perceived stress (PSS-10), mean (sum score)^a^	10	1.10 (11)	4.60 (46)	2.75 (27.41)	0.68 (6.82)	0.89
***Post-intervention***						
Source credibility^a^	3	2.00	7.00	5.25	0.96	0.76
Perceived similarity^b^	5	1.00	7.00	4.21	1.19	0.88
Intentions (UTAUT, post)^a^	3	1.00	7.00	4.72	1.15	0.73
Attitude (PU, post)^a^	3	1.33	7.00	5.38	0.91	0.86
Attitude, clients (APOI)^a^	16	1.88	4.31	3.18	0.52	0.82
Attitude, public (ETAM)^a^	17	1.29	5.00	3.16	0.57	0.87

a *N=231 (full sample, four groups)*.

b *n=176 (intervention groups only, three groups)*.

*Min., minimum; Max., maximum; SD, standard deviation; APOI, Attitudes toward Psychological Online Interventions (attitudes toward online interventions such as online therapies by clients; clinical context); ETAM*, *E-Therapy Measure (public attitudes toward online psychotherapy); post, post-intervention assessment; PSS-10, Perceived Stress Scale, 10 items (adapted scale range: 1–5); PU, perceived usefulness; UTAUT, Unified Theory of Acceptance and Use of Technology*.

At baseline, participants were asked to answer few background questions (e.g., age, gender, study model, and experience with digi-MHSs). Next, baseline attitudes and intentions regarding digi-MHSs were measured using three items each on a response scale ranging from 1 (“fully disagree”) to 7 (“fully agree”). Specifically, we assessed behavioral intentions based on the *Unified Theory of Acceptance and Use of Technology* (UTAUT; Venkatesh et al., 2003) using a German adaptation (Hennemann et al., 2016), while the attitude short scale emphasizing perceived usefulness (PU) was grounded on the *Theory of Planned Behavior* (TPB; Ajzen, 1991) and pretested in previous work (Apolinário-Hagen et al., 2021). Perceived stress in the past 2weeks was measured with the validated 10-items German version of the *Perceived Stress Scale* (PSS-10; Klein et al., 2016) on a Likert scale ranging from 1 (“never”) to 5 (“very often”; adapted scale sum range: 10–50).

Next, participants were automatically randomized to one of four information groups, either the aCG (“information only”) or one of three IGs: IG1 (employees), IG2 (university students), or IG3 (experts), each receiving different additional testimonials on an online stress management training, as documented in Supplementary Material 1.

At post-intervention, we assessed the mediators perceived similarity (five items, IGs only) and source credibility (three items, all four groups) in line with a pilot trial (Apolinário-Hagen et al., 2021). Attitudes and intentions were measured again with the short scales described above. To extend the scope to further digi-MHS applications, attitudes toward online psychotherapies were assessed on a Likert scale with 1 (“fully disagree”) to 5 (“fully agree”) using the scales *Attitudes to Psychological Online Interventions* (APOI; Schröder et al., 2015) with 16 items (eight inverted items) and the *E-Therapy Attitude Measure* (ETAM; Apolinário-Hagen et al., 2018b) with 17 items. Finally, all participants were debriefed.

### Statistical Analyses

Collected data were extracted from *Unipark*, if marked as completed or screened out. Data handling was done in accordance with a pilot trial (Apolinário-Hagen et al., 2021), as documented in Figure S1 in the Supplementary Material 2. Imputation by mean was performed in case of few missing values (e.g., one missing value in the ETAM). Research questions were tested on an alpha level of 0.05 (two-fold) using IBM-SPSS, version 26.

We conducted independent two-sample *t*-tests to compare the aCG with the IGs (RQ1) in attitudes and intentions at post-intervention (added value of testimonials, coding: aCG=0, IGs=1). Furthermore, we performed one-way ANOVA to determine differences in attitudes and intentions between the four study arms (RQ2; differentiated effects of testimonial sources) at post-intervention, including *post-hoc* tests (Bonferroni, multiple comparisons) and the adjustment of baseline values (ANCOVA for sensitivity analyses). Effect sizes were classified according to social sciences’ conventions (Cohen, 1988).

Mediation analyses (RQ3) were performed using the *PROCESS* macro for SPSS by Hayes, version 3.4 (Hayes, 2018), with source credibility and perceived similarity as mediators, testimonial type [dummy-coding: IG1 versus IG2 (IG1=0, IG2=1), IG2 versus IG3 (IG2=0, IG3=1), IG1 versus IG3 (IG1=0, IG3=1)] as independent and attitudes as well as intentions as dependent variables (5,000 bootstrapping samples).

## Results

### Participant Characteristics

Out of *n*=231 included university students, *n*=55 were randomly assigned to the aCG, while the other were additionally presented either with testimonials by employees (IG1, *n*=60), university students (IG2, *n*=58), or experts (IG3, *n*=58). The median age was 29years (*Mean*=32.02, *SD*=10.5, range 18–62years). Most (85.3%) were women (14.3% men, 0.4% other). Recruitment sources were 58.9% the University of Hagen (e.g., *virtual lab*), 22.9% *via* Facebook and 18.2% *via* other options.

With 46.3%, the majority reported the general university entrance qualification as highest educational attainment, followed by 26.0% with a bachelor’s degree, 15.6% with a master’s degree, and 12.1% with other qualifications. Most (66.2%) indicated distance learning university as study model, while 26.9% were enrolled in a traditional university and 6.5% in both study models simultaneously (other: 0.4%). Sixty-one percent (*n*=141) studied full-time and 39.0% (*n*=90) in part-time.

### Descriptive and Ancillary Analyses

Regarding awareness, 26.8% (*n*=62) of the sample reported to have heard about digi-MHSs [“no”: *n*=155 (67.1%); “not sure”: *n*=14 (6.1%)], while 7.8% (*n*=18) stated to have obtained more information on specific digi-MHSs and 4.8% (*n*=11) indicated respective experience.

Table 1 shows psychometric data of the assessed scales. Descriptive data differentiated by experimental group and ancillary analyses can be found in the Supplementary Material 2. For instance, perceived stress was moderately high according the *PSS-10*, but only weakly correlated with intentions (*r*=0.159, *p*=0.015) and attitudes (*ETAM*; *r*=0.161, *p*=0.014) at post-intervention.

### Main Outcomes on Group Differences

#### RQ1: Benefits of Adding Testimonials to Information

**RQ1a:** Regarding attitudes, as measured with the short scale, we found a significant difference [*t*_(229)_=−2.06, *p*=0.041], with less favorable attitudes in the aCG (*Mean*=5.16, *SD*=0.99, *n*=55) compared to the IGs (*Mean*=5.45, *SD*=0.87, *n*=176), *d*=0.32. In contrast, we found no differences in attitudes toward online therapies according to the *APOI* [*t*_(229)_=0.20, *p*=0.839] and *ETAM* [*t*_(229)_=−1.05, *p*=0.296].

**RQ1b:** Intentions to use digi-MHSs were significantly lower in the aCG (*Mean*=4.29, *SD*=1.15, *n*=55) than in the IGs (*Mean*=4.86, *SD*=1.12, n=176) at post-intervention, *t*_(229)_=−3.26, *p*=0.001, *d*=0.50.

#### RQ2: Differences Between Information Sources

**RQ2a:** Regarding attitudes, we found no significant differences between the study arms, as measured by the short scale at post-intervention [one-way ANOVA, *F*_(3, 227)_=2.23, *p*=0.086] regarding digi-MHSs for health promotion [after adjusting for baseline attitude: *F*_(3, 226)_=1.27, *p*=0.285, $\eta_{p}^{2}$ =0.017], and online therapies according to the *APOI* [*F*_(3, 227)_=1.51, *p*=0.213] and *ETAM* [*F*_(3, 227)_=1.29, *p*=0.279].

**RQ2b:** One-way ANOVA demonstrated differences in intentions to use digi-MHSs between the study arms before [*F*_(3,227)_=11.48, *p*<0.001, $\eta_{p}^{2}$ =0.13] and after adjusting for baseline intentions [ANCOVA, *F*_(3, 226)_=23.92, *p*<0.001, $\eta_{p}^{2}$ =0.24].

As shown in Table 2, Bonferroni-adjusted *post-hoc* tests of the ANCOVA revealed higher intentions to use digi-MHSs only after exposure to students’ testimonials (*p*s<0.001).

**Table 2:** Differences between the experimental groups in intentions to use digital mental health interventions at post-intervention after adjusting for baseline intentions (multiple comparisons).

					95% CI	
(I)	(J)	∆ *M* (I-J)	*SD*	*p*	*LL*	*UL*
aCG	IG1	−0.143	0.124	1.00	−0.474	0.188
(control)	IG2	−0.903^***^	0.124	<0.001	−1.233	−0.573
	IG3	−0.047	0.124	1.00	−0.377	0.282
IG1	aCG	0.143	0.124	1.00	−0.188	0.474
(staff)	IG2	−0.760^***^	0.122	<0.001	−1.084	−0.436
	IG3	0.096	0.122	1.00	−0.230	0.422
IG2	aCG	0.903^***^	0.124	<0.001	0.573	1.233
(student)	IG1	0.760^***^	0.122	<0.001	0.436	1.084
	IG3	0.856^***^	0.122	<0.001	0.530	1.181
IG3	aCG	0.047	0.124	1.00	−0.282	0.377
(expert)	IG1	−0.096	0.122	1.00	−0.422	0.230
	IG2	−0.856^***^	0.122	<0.001	−1.181	−0.530

*** *p<0.001*.

*N=231. Post-hoc tests with Bonferroni adjustment, dependent variable: intentions to use digital mental health services (digi-MHSs) for stress management purposes, independent variable: study group (information intervention), covariate: baseline intentions. ∆ M, Mean difference; SD, Standard deviation; 95% CI, 95% confidence interval; LL, lower limits; UL, upper limits. Abbreviations (study arms): aCG, active control group (“information only”); IG, narrative intervention group (IG); IG1, information plus testimonials by unspecified staff; IG2, information plus testimonials by university students; IG3, information plus testimonials by experts*.

#### RQ3: Mediation Effects

##### RQ3a: Perceived Similarity

As shown in Table 3, perceived similarity fully mediated the effect of testimonials for students (IG1) versus employees (IG2) on intention to use [indirect effect=0.289, 95% CI (0.100, 0.528)], as well as on attitudes toward digi-MHSs [indirect effect=0.212, 95% CI (0.030, 0.441)].

**Table 3:** Mediation analyses on the influence of narrative intervention type on acceptance.

IV	DV	Mediator	*a*	*b*	*ab*	Indirect effect		*c′*	Direct effect	
						95% CI_ab_		*X-Y*	95% CI_c′_	
*X*	*Y*	*M*	*X-M*	*M-Y*	*X-M-Y*	*LL*	*UL*		*LL*	*UL*
IG1-IG2	Intention	Similarity	0.933^***^	0.310^***^	0.289	0.100	0.528+	0.232	−0.0164	0.629
		Credibility	0.009	0.259^*^	0.002	−0.070	0.068	0.232	−0.164	0.629
	Attitude	Similarity	0.933^***^	0.205^**^	0.212	0.030	0.441+	−0.121	−0.439	0.197
		Credibility	0.009	0.392^***^	0.004	−0.131	0.135	−0.121	−0.439	0.197
IG1-IG3	Intention	Similarity	−0.164	0.353^***^	−0.058	−0.214	0.094	−0.304	−0.648	0.040
		Credibility	−0.330	0.280^**^	−0.070	−0.189	0.006	−0.304	−0.648	0.040
	Attitude	Similarity	−0.164	0.218^**^	−0.029	−0.113	0.055	−0.093	−0.401	0.215
		Credibility	−0.330	0.290^***^	−0.096	−0.225	0.007	−0.093	−0.401	0.215
IG3-IG2	Intention	Similarity	−1.10^***^	0.332^**^	−0.364	−0.651	−0.101+	−0.534	−0.956	−0.113
		Credibility	−0.340^*^	0.163	−0.055	−0.170	0.026	−0.534	−0.956	−0.113
	Attitude	Similarity	−1.10^***^	0.095	−0.104	−0.249	0.023	−0.223	−0.336	0.291
		Credibility	−0.340^*^	0.304^***^	−0.103	−0.217	−0.004+	−0.223	−0.336	0.291

* *p<0.05*,

** *p<0.01*,

*** *p<0.001*.

*a=effect of X (IV, independent variable, intervention) on M (mediator), b=effect of the mediator M on Y (DV, dependent variable; attitude or intentions in terms of acceptance), ab=indirect effect via the mediator M, c′=direct effect of X (IV) on Y (DV); 95% CI, 95% confidence interval; LL, lower limits; UL, upper limits; + means that the confidence interval of the ab-path does not include the number zero (i.e., significant indirect effect). Subsample: n=116 (IG3 versus IG2), n=118 (IG1 versus IG2). Abbreviations concerning the narrative information groups: IG, intervention group (i.e., information with added testimonials), IG1=information plus testimonials by staff (i.e., employees with unspecified occupation, working outside of academia as work area), IG2=information plus testimonials by university students, IG3=information plus testimonials by experts*.

In addition, there was a partial mediation for perceived similarity in IG3 versus IG2 [indirect effect=−0.364, 95% CI (−0.651, −0.101)], with higher intentions in case of greater similarity following the exposure to testimonials by students compared to experts.

##### RQ3b: Source Credibility

Source credibility fully mediated the influence of students’ testimonials on attitudes in comparison to expert testimonials [IG2 versus IG3, indirect effect=−0.103, 95% CI (−0.217, −0.004)]. There was no mediation effect of source credibility, neither on attitudes in comparison of IG1 versus IG3 (staff versus expert) nor on intentions.

## Discussion

This study aimed to explore the influence of testimonials on the acceptance of digi-MHS among university students as well as mediation effects.

### RQ1: Added Value of Testimonials

Concerning the efficacy of narrative interventions, our analyses showed that the exposure to testimonials in addition to written information was associated with higher intentions to use (*d*=0.50) and more positive attitudes toward digi-MHSs for stress prevention (*d*=0.32), compared to mere information. Hence, this study indicated that the acceptance of digi-MHS for stress management can be improved to a meaningful extent by a simple testimonial intervention. This finding corresponds to prior work on acceptance-facilitating interventions involving multi-component information on digi-MHSs, like iCBT (Ebert et al., 2015; Soucy et al., 2016). In contrast, we found no testimonial effects on attitudes toward online therapies, which is potentially due to the scope on health promotion in the stimulus material. Overall, however, the evidence base for narrative interventions is indecisive (Shaffer et al., 2018) and particularly scarce for mental health services. Consequently, the identified testimonial effects in digital mental health promotion can be considered as one major contribution of this experiment.

### RQ2: Differences in Acceptance

Another goal was to compare the influence of different information types on the acceptance of digi-MHSs. We identified higher intentions attributable only to students’ testimonials compared to each other information group before and after adjusting for baseline intention values, with moderate-to-high effect size. While the influence of students’ testimonials appears plausible, it was unexpected to identify no influence of expert statements. Potentially, expert testimonials were processed rather more analytically than first-person testimonials. Participants may have concluded that these testimonial sources intended to persuade them, which may have led to less trustworthiness and more reactance (Wang and Shen, 2019). Accordingly, a recent survey indicated that recipients of health advertisements were concerned regarding the inappropriate use of academic reputation (doctors as expert sources) and found that testimonials should be viewed more critically in healthcare compared to consumer contexts (Holden et al., 2021). To date, only few investigations on effects of expert versus lay people testimonials on digi-MHSs exist and yielded indecisive results (Healey et al., 2017). Here, we confirmed positive influences of first-person testimonials, which have been shown to be more persuasive than third-person narratives in other health promotion experiments (Chen and Bell, 2021).

In contrast to intentions, we found no group difference in attitudes toward online interventions for stress coping or therapy. Possibly, attitudes were easier biased by social desirability than intentions, making it more difficult to induce improvements with source-related differences. In addition, attitudes and intentions represent different stages of adoption in terms of intention as mediator of the effect of attitude on behavior in line with the TPB (Ajzen, 1991; MacKinnon et al., 2000). According to a meta-analysis, research showed that statistical evidence seems to be more suitable to improve attitudes and beliefs (cognitive elaboration) and that narrative messages rather influence affective responses, like behavioral intentions (Zebregs et al., 2015). However, none of the reviewed studies focused on mental health or eHealth. Therefore, more research is required to explore source-related effects of testimonials and related factors not only on the acceptance of digi-MHSs but also on the influence of acceptance on subsequent registration or uptake rates (Healey et al., 2017; Wopperer et al., 2019) as well as successful program completions (Fleming et al., 2018).

Recent research demonstrated an association between prior experience with digi-MHSs and higher acceptance among university students, but also little experience and low uptake rates at the same time (Lavergne and Kennedy, 2021). Additionally, previous experience appears not mandatory to form positive attitudes toward digi-MHSs (Mayer et al., 2019). Overall, the low experience rates regarding digi-MHSs in our sample (5%) correspond to earlier surveys from Germany (Webelhorst et al., 2020; Breil et al., 2021) and international findings across different populations (Toscos et al., 2018; Clough et al., 2019; Richardson et al., 2020). Although research has revealed positive attitudes and the readiness to try stand-alone digi-MHSs among university students (Hadler et al., 2021), in direct comparison face-to-face support, including blended care, have been shown to be preferred in surveys, including discrete choice experiments (Phillips et al., 2021). Future experiments on acceptance-facilitating interventions may therefore extend the scope to blended interventions.

### RQ3: Mediation Effects

Another purpose was to identify mediators of attitudes and intentions. Consistent with prior work (Apolinário-Hagen et al., 2021), perceived similarity mediated the influence of exposure to testimonials on attitudes toward and intentions to use digi-MHSs for mental health promotion, favoring students’ over employees’ testimonials (IG1 versus IG2). In addition, there was a full mediation effect of similarity on intentions (students’ versus expert testimonials). Thus, the acceptance-facilitating role of similarity seems to be a promising future focus when designing information aiming at promoting the adoption of digi-MHSs.

Furthermore, we found a full mediation effect of source credibility on attitudes, with student testimonials being assessed as more credible than those by experts (IG2 versus IG3). In contrast, there were no mediation effects of source credibility on intentions. Interestingly, there were no differences between the IGs in source credibility, while the aCG assessed the information significantly as more credible than participants receiving expert testimonials. Potentially, the expert testimonials were not optimally designed, could have been presented by recognized experts and additionally integrated critical statements. Increasing credibility can be a starting point, which may be promoted by certification and quality seals. Since fall 2020, the Digital Healthcare Act allows for the prescription of certified health apps in Germany (Gerke et al., 2020). Yet, many concerns persist among health professionals, especially regarding data security (Heidel and Hagist, 2020). Future studies on acceptance-facilitating interventions could therefore focus on balancing information on the benefits (safety, effectiveness) with contraindications of quality-approved mental health apps.

### Limitations

Limitations of this study include multiple testing (i.e., risk of false positive findings, “p-hacking”), fictional, positively framed testimonials and unequal group sizes for testing the effects of testimonials in RQ1.

Out of *n*=368 data sets, *n*=184 (37%) were removed mostly due to withdrawal of consent, dropping out prior to randomization or unrealistic participation time, as shown in Figure S1 (Supplementary Material 2). Nonetheless, this rate corresponds to other online studies using the *virtual lab* (Apolinário-Hagen et al., 2021).

Due to the integration in a Master thesis project, the recruitment period was limited and scheduled in the early winter semester 2020/21. Furthermore, we did neither measure the semester nor mental health status, except for stress, to reduce the amount of identifiable or sensitive data.

In addition, it may also have been useful to repeat health messages to achieve more robust persuasive effects (Suka et al., 2020). Furthermore, we did not include information on the costs of eMHSs to reduce the amount of attributes and given the universally free access to healthcare in Germany.

Finally, it should be considered that about two-third of the sample were distance-learning students who differ from traditional students in demographic background and study conditions (Harrer et al., 2021), whereas the Covid-19 pandemic contributed to at least comparable distance study conditions in fall 2020. Moreover, online education and the unavailability of face-to-face support may have had an impact on the acceptance of digi-MHSs.

## Conclusion

Taken together, this experiment identified positive influences of first-person testimonials on the acceptance of digi-MHS among university students, indicating that even such simple narrative interventions may be an option for information campaigns. Specifically, program information supplemented with students’ testimonials could be useful in increasing behavioral intentions. In a next step, the most relevant domains for fostering perceived similarity and credibility could be explored in more detail. Further insights into these mediating effects on acceptance may help develop tailored information on digi-MHSs.

## Data Availability Statement

The datasets presented in this study can be found in online repositories. The names of the repository/repositories and accession number(s) can be found at: https://doi.org/10.7802/2287.

## Ethics Statement

The studies involving human participants were reviewed and approved by Ethics committee of the University of Hagen, Germany (EA_278_2020). The patients/participants provided their written informed consent to participate in this study.

## Author Contributions

JA-H conceived the study idea and study design, initiated the study, wrote the first draft of the manuscript, and coordinated and finalized the article. LF and JW sought ethical approval. CS, JW, LF, MH, FW, DE, and DL made relevant contributions to the study design and interpretation of data. JW programmed the online questionnaire, recruited participants, and collected and analyzed data under supervision of LF and CS within JW’s master thesis project. FW and JA-H cross-checked the data underlying the manuscript and prepared the data set for sharing for non-commercial purposes. All authors read the manuscript, provided feedback, and approved the final version.

## Conflict of Interest

DE is a shareholder, and DL and MH are minor shareholders of the Institute for health trainings online (HelloBetter), which aims to implement scientific findings related to health interventions into routine care. DE reports to have received consultancy fees or served in the scientific advisory board from several companies, such as Novartis, Sanofi, digital Lantern, Schön Kliniken, Minddistrict, and German health insurance companies (BARMER, Techniker Krankenkasse).

The remaining authors declare that the research was conducted in the absence of any commercial or financial relationships that could be construed as a potential conflict of interest.

## Publisher’s Note

All claims expressed in this article are solely those of the authors and do not necessarily represent those of their affiliated organizations, or those of the publisher, the editors and the reviewers. Any product that may be evaluated in this article, or claim that may be made by its manufacturer, is not guaranteed or endorsed by the publisher.

## Abbreviations

aCG: active control group (information only)
APOI: attitudes toward psychological online interventions (questionnaire)
digi-MHSs: digital mental health services
ETAM: e-therapy attitude measure
IG: intervention group (receiving information plus testimonials)
PSS: perceived stress scale
PU: perceived usefulness (attitude short scale)
RQ: research question
TPB: theory of planned behavior
UTAUT: unified theory of acceptance and use of technology

## References

[ref1] Ajzen I. (1991). The theory of planned behavior. Organ. Behav. Hum. Decis. Process. 50, 179–211. doi: 10.1016/0749-5978(91)90020-T

[ref2] Apolinário-Hagen J. Fritsche L. Bierhals C. Salewski C. (2018a). Improving attitudes toward e-mental health services in the general population via psychoeducational information material. Internet Interv. 12, 141–149. doi: 10.1016/j.invent.2017.12.002, PMID: 30135778 PMC6096329

[ref3] Apolinário-Hagen J. Harrer M. Dederichs M. Fritsche L. Wopperer J. Wals F.. (2021). Exploring the influence of testimonial source on attitudes towards e-mental health interventions among university students: Four-group randomized controlled trial. PLoS One 16:e0252012. doi: 10.1371/journal.pone.0252012, PMID: 34038455 PMC8153476

[ref4] Apolinário-Hagen J. Harrer M. Kählke F. Fritsche L. Salewski C. Ebert D. D. (2018b). Public attitudes toward guided internet-based therapies: web-based survey study. JMIR Ment. Health 5:e10735. doi: 10.2196/10735, PMID: 29764797 PMC5974457

[ref5] Auerbach R. P. Mortier P. Bruffaerts R. Alonso J. Benjet C. Cuijpers P.. (2018). WHO world mental health surveys international college student project: prevalence and distribution of mental disorders. J. Abnorm. Psychol. 127, 623–638. doi: 10.1037/abn0000362, PMID: 30211576 PMC6193834

[ref6] Benjet C. (2020). Stress management interventions for college students in the context of the COVID-19 pandemic. Clin. Psychol. Sci. Pract.:e12353. [Epub ahead of print]. doi: 10.1111/cpsp.12353, PMID: 32837028 PMC7323064

[ref7] Breil B. Dederichs M. Kremer L. Richter D. Angerer P. Apolinário-Hagen J. (2021). Bekanntheit und Nutzung von digitalen Gesundheitsangeboten in Deutschland: eine bevölkerungsrepräsentative Querschnittsuntersuchung [Awareness and use of digital health services in Germany: a cross-sectional study representative of the population]. Das Gesundheitswesen. [Epub ahead of print]. doi: 10.1055/a-1335-4245, PMID: 33862648

[ref8] Chaiken S. (1980). Heuristic versus systematic information processing and the use of source versus message cues in persuasion. J. Pers. Soc. Psychol. 39, 752–766. doi: 10.1037/0022-3514.39.5.752

[ref9] Chen M. Bell R. A. (2021). A meta-analysis of the impact of point of view on narrative processing and persuasion in health messaging. Psychol. Health, 1–18. doi: 10.1080/08870446.2021.1894331, PMID: [Epub ahead of print] 33678078

[ref10] Clough B. A. Zarean M. Ruane I. Mateo N. J. Aliyeva T. A. Casey L. M. (2019). Going global: do consumer preferences, attitudes, and barriers to using e-mental health services differ across countries? J. Ment. Health 28, 17–25. doi: 10.1080/09638237.2017.1370639, PMID: 28857650

[ref11] Cohen J. (1988). Statistical Power Analysis for the Behavioral Sciences. 2nd Edn. Hillsdale, NJ: Erlbaum.

[ref12] Cuijpers P. Auerbach R. P. Benjet C. Bruffaerts R. Ebert D. Karyotaki E.. (2019). The World Health Organization world mental health international college student initiative: an overview. Int. J. Methods Psychiatr. Res. 28:e1761. doi: 10.1002/mpr.1761, PMID: 30614123 PMC6590455

[ref13] Cunningham C. E. Walker J. R. Eastwood J. D. Westra H. Rimas H. Chen Y.. (2014). Modeling mental health information preferences during the early adult years: a discrete choice conjoint experiment. J. Health Commun. 19, 413–440. doi: 10.1080/10810730.2013.811324, PMID: 24266450 PMC3996536

[ref14] Cunningham C. E. Zipursky R. B. Christensen B. K. Bieling P. J. Madsen V. Rimas H.. (2017). Modeling the mental health service utilization decisions of university undergraduates: A discrete choice conjoint experiment. J. Am. Coll. Heal. 65, 389–399. doi: 10.1080/07448481.2017.1322090, PMID: 28511031

[ref15] Dadaczynski K. Okan O. Messer M. Leung A. Y. M. Rosário R. Darlington E.. (2021). Digital health literacy and web-based information-seeking behaviors of university students in Germany during the COVID-19 pandemic: cross-sectional survey study. J. Med. Internet Res. 23:e24097. doi: 10.2196/24097, PMID: 33395396 PMC7813561

[ref16] Davenport T. A. Cheng V. W. S. Iorfino F. Hamilton B. Castaldi E. Burton A.. (2020). Flip the clinic: a digital health approach to youth mental health service delivery during the COVID-19 pandemic and beyond. JMIR Ment. Health 7:e24578. doi: 10.2196/24578, PMID: 33206051 PMC7744139

[ref17] Dederichs M. Weber J. Pischke C. R. Angerer P. Apolinário-Hagen J. (2021). Exploring medical students’ views on digital mental health interventions: A qualitative study. Internet Interv. 25:100398. doi: 10.1016/j.invent.2021.100398, PMID: 34026567 PMC8122007

[ref18] Dunbar M. S. Sontag-Padilla L. Kase C. A. Seelam R. Stein B. D. (2018). Unmet mental health treatment need and attitudes toward online mental health services among community college students. Psychiatr. Serv. 69, 597–600. doi: 10.1176/appi.ps.201700402, PMID: 29540117

[ref19] Ebert D. D. Berking M. Cuijpers P. Lehr D. Pörtner M. Baumeister H. (2015). Increasing the acceptance of internet-based mental health interventions in primary care patients with depressive symptoms. A randomized controlled trial. J. Affect. Disord. 176, 9–17. doi: 10.1016/j.jad.2015.01.056, PMID: 25682378

[ref20] Ebert D. D. Franke M. Kählke F. Küchler A.-M. Bruffaerts R. Mortier P.. (2018). Increasing intentions to use mental health services among university students. Results of a pilot randomized controlled trial within the World Health Organization’s world mental health international college student initiative. Int. J. Methods Psychiatr. Res. 28:e1754. doi: 10.1002/mpr.1754, PMID: 30456814 PMC6877244

[ref21] Ebert D. D. Harrer M. Apolinário-Hagen J. Baumeister H. (2019). Digital interventions for mental disorders: key features, efficacy, and potential for artificial intelligence applications. Adv. Exp. Med. Biol. 1192, 583–627. doi: 10.1007/978-981-32-9721-0_29, PMID: 31705515

[ref22] Ebert D. D. Lehr D. Heber E. Riper H. Cuijpers P. Berking M. (2016). Internet- and mobile-based stress management for employees with adherence-focused guidance: efficacy and mechanism of change. Scand. J. Work Environ. Health 42, 382–394. doi: 10.5271/sjweh.3573, PMID: 27249161

[ref23] Faul F. Erdfelder E. Lang A.-G. Buchner A. (2007). G*Power 3. Behav. Res. Methods 39, 175–191. doi: 10.3758/BF03193146, PMID: 17695343

[ref24] Fleming T. Bavin L. Lucassen M. Stasiak K. Hopkins S. Merry S. (2018). Beyond the trial: systematic review of real-world uptake and engagement with digital self-help interventions for depression, low mood, or anxiety. J. Med. Internet Res. 20:e199. doi: 10.2196/jmir.9275, PMID: 29875089 PMC6010835

[ref25] Gerke S. Stern A. D. Minssen T. (2020). Germany’s digital health reforms in the COVID-19 era: lessons and opportunities for other countries. NPJ Digit. Med. 3:94. doi: 10.1038/s41746-020-0306-7, PMID: 32685700 PMC7351985

[ref26] Gigerenzer G. Gaissmaier W. (2011). Heuristic decision making. Annu. Rev. Psychol. 62, 451–482. doi: 10.1146/annurev-psych-120709-145346, PMID: 21126183

[ref27] Green M. C. Clark J. L. (2013). Transportation into narrative worlds: implications for entertainment media influences on tobacco use. Addiction 108, 477–484. doi: 10.1111/j.1360-0443.2012.04088.x, PMID: 22994374

[ref28] Hadler N. L. Bu P. Winkler A. Alexander A. W. (2021). College student perspectives of telemental health: a review of the recent literature. Curr. Psychiatry Rep. 23:6. doi: 10.1007/s11920-020-01215-7, PMID: 33404975 PMC7785477

[ref29] Harrer M. Adam S. H. Baumeister H. Cuijpers P. Karyotaki E. Auerbach R. P.. (2018). Internet interventions for mental health in university students: A systematic review and meta-analysis. Int. J. Methods Psychiatr. Res. 28:e1759. doi: 10.1002/mpr.1759, PMID: 30585363 PMC6877279

[ref30] Harrer M. Apolinário-Hagen J. Fritsche L. Salewski C. Zarski A.-C. Lehr D.. (2021). Effect of an internet- and app-based stress intervention compared to online psychoeducation in university students with depressive symptoms: Results of a randomized controlled trial. Internet Interv. 24:100374. doi: 10.1016/j.invent.2021.100374, PMID: 33718001 PMC7932886

[ref31] Hayes A. F. (2018). Introduction to Mediation, Moderation, and Conditional Process Analysis: A Regression-Based Approach. 2nd Edn. New York, London: The Guilford Press.

[ref32] Healey B. J. Griffiths K. M. Bennett K. (2017). The effect of programme testimonials on registrations for an online cognitive behaviour therapy intervention. A randomised trial. Digit. Health 3:205520761772993. doi: 10.1177/2055207617729937, PMID: 29942614 PMC6001272

[ref33] Heidel A. Hagist C. (2020). Potential benefits and risks resulting from the introduction of health apps and wearables into the German statutory health care system: scoping review. JMIR Mhealth Uhealth 8:e16444. doi: 10.2196/16444, PMID: 32965231 PMC7542416

[ref34] Hennemann S. Beutel M. E. Zwerenz R. (2016). Drivers and barriers to acceptance of web-based aftercare of patients in inpatient routine care. J. Med. Internet Res. 18:e337. doi: 10.2196/jmir.6003, PMID: 28011445 PMC5219589

[ref35] Holden A. Nanayakkara S. Skinner J. Spallek H. Sohn W. (2021). What do Australian health consumers believe about commercial advertisements and testimonials? A survey on health service advertising. BMC Public Health 21:74. doi: 10.1186/s12889-020-10078-9, PMID: 33413201 PMC7791787

[ref36] Klein E. M. Brähler E. Dreier M. Reinecke L. Müller K. W. Schmutzer G.. (2016). The German version of the Perceived Stress Scale – psychometric characteristics in a representative German community sample. BMC Psychiatry 16:159. doi: 10.1186/s12888-016-0875-9, PMID: 27216151 PMC4877813

[ref37] Kohls E. Baldofski S. Moeller R. Klemm S.-L. Rummel-Kluge C. (2021). Mental health, social and emotional well-being, and perceived burdens of university students during COVID-19 pandemic lockdown in Germany. Front. Psychiatry. 12:643957. doi: 10.3389/fpsyt.2021.643957, PMID: 33889102 PMC8055863

[ref38] Larsen M. E. Huckvale K. Nicholas J. Torous J. Birrell L. Li E.. (2019). Using science to sell apps: evaluation of mental health app store quality claims. NPJ Digit. Med. 2:18. doi: 10.1038/s41746-019-0093-1, PMID: 31304366 PMC6550255

[ref39] Lattie E. G. Adkins E. C. Winquist N. Stiles-Shields C. Wafford Q. E. Graham A. K. (2019). Digital mental health interventions for depression, anxiety, and enhancement of psychological well-being among college students: systematic review. J. Med. Internet Res. 21:e12869. doi: 10.2196/12869, PMID: 31333198 PMC6681642

[ref40] Lavergne J. A. Kennedy M. L. (2021). Telepsychiatry and medical students: a promising mental health treatment for medical student use both personally and professionally. Curr. Psychiatry Rep. 23:31. doi: 10.1007/s11920-021-01248-6, PMID: 33851272 PMC8043086

[ref41] Machleid F. Kaczmarczyk R. Johann D. Balčiūnas J. Atienza-Carbonell B. Maltzahn F. von. (2020). Perceptions of digital health education among European medical students: mixed methods survey. J. Med. Internet Res. 22:e19827. doi: 10.2196/19827, PMID: 32667899 PMC7455864

[ref42] MacKinnon D. P. Krull J. L. Lockwood C. M. (2000). Equivalence of the mediation, confounding and suppression effect. Prev. Sci. 1, 173–181. doi: 10.1023/A:1026595011371, PMID: 11523746 PMC2819361

[ref43] Mayer G. Gronewold N. Alvarez S. Bruns B. Hilbel T. Schultz J.-H. (2019). Acceptance and expectations of medical experts, students, and patients toward electronic mental health apps: cross-sectional quantitative and qualitative survey study. JMIR Ment. Health 6:e14018. doi: 10.2196/14018, PMID: 31763990 PMC6902133

[ref44] Montagni I. Tzourio C. Cousin T. Sagara J. A. Bada-Alonzi J. Horgan A. (2020). Mental health-related digital use by university students: a systematic review. Telemed. e-Health 26, 131–146. doi: 10.1089/tmj.2018.0316, PMID: 30888256

[ref45] Perrier M.-J. Martin Ginis K. A. (2017). Narrative interventions for health screening behaviours: a systematic review. J. Health Psychol. 22, 375–393. doi: 10.1177/1359105315603463, PMID: 26359288

[ref46] Petty R. E. Barden J. Wheeler S. C. (2009). “The elaboration likelihood model of persuasion: developing health promotions for sustained behavioral change,” in Emerging Theories in Health Promotion Practice and Research. eds. DiClemente R. J. Crosby R. A. Kegler M. C. (San Francisco, CA, US: Jossey-Bass/Wiley), 185–214.

[ref47] Phillips E. A. Himmler S. F. Schreyögg J. (2021). Preferences for e-mental health interventions in Germany: a discrete choice experiment. Value Health 24, 421–430. doi: 10.1016/j.jval.2020.09.018, PMID: 33641777

[ref48] Pruitt L. D. Zoellner L. A. Feeny N. C. Caldwell D. Hanson R. (2012). The effects of positive patient testimonials on PTSD treatment choice. Behav. Res. Ther. 50, 805–813. doi: 10.1016/j.brat.2012.09.007, PMID: 23103234 PMC3519362

[ref49] Quintero Johnson J. M. Sangalang A. Park S.-Y. (2021). First-person, third-person, or bystander? Exploring the persuasive influence of perspective in mental health narratives. J. Health Commun. 26, 225–238. doi: 10.1080/10810730.2021.1916658, PMID: 33910481

[ref50] Quintero Johnson J. M. Yilmaz G. Najarian K. (2017). Optimizing the presentation of mental health information in social media: the effects of health testimonials and platform on source perceptions, message processing, and health outcomes. Health Commun. 32, 1121–1132. doi: 10.1080/10410236.2016.1214218, PMID: 27573748

[ref51] Richardson C. G. Slemon A. Gadermann A. McAuliffe C. Thomson K. Daly Z.. (2020). Use of asynchronous virtual mental health resources for COVID-19 pandemic-related stress among the general population in Canada: cross-sectional survey study. J. Med. Internet Res. 22:e24868. doi: 10.2196/24868, PMID: 33315583 PMC7775378

[ref52] Schröder J. Sautier L. Kriston L. Berger T. Meyer B. Späth C.. (2015). Development of a questionnaire measuring Attitudes towards Psychological Online Interventions-the APOI. J. Affect. Disord. 187, 136–141. doi: 10.1016/j.jad.2015.08.044, PMID: 26331687

[ref53] Shaffer V. A. Focella E. S. Hathaway A. Scherer L. D. Zikmund-Fisher B. J. (2018). On the usefulness of narratives: an interdisciplinary review and theoretical model. Ann. Behav. Med. 52, 429–442. doi: 10.1093/abm/kax008, PMID: 29684135 PMC6369912

[ref54] Shaffer V. A. Zikmund-Fisher B. J. (2013). All stories are not alike: a purpose-, content-, and valence-based taxonomy of patient narratives in decision aids. Med. Decis. Mak. 33, 4–13. doi: 10.1177/0272989X12463266, PMID: 23065418

[ref55] Shen F. Sheer V. C. Li R. (2015). Impact of narratives on persuasion in health communication: a meta-analysis. J. Advert. 44, 105–113. doi: 10.1080/00913367.2015.1018467

[ref56] Soucy J. N. Owens V. A. M. Hadjistavropoulos H. D. Dirkse D. A. Dear B. F. (2016). Educating patients about internet-delivered cognitive behaviour therapy: Perceptions among treatment seekers and non-treatment seekers before and after viewing an educational video. Internet Interv. 6, 57–63. doi: 10.1016/j.invent.2016.09.003, PMID: 30135814 PMC6096293

[ref57] Suka M. Yamauchi T. Yanagisawa H. (2020). Persuasive messages can be more effective when repeated: A comparative survey assessing a message to seek help for depression among Japanese adults. Patient Educ. Couns. 103, 811–818. doi: 10.1016/j.pec.2019.11.008, PMID: 31761527

[ref58] Toscos T. Carpenter M. Drouin M. Roebuck A. Kerrigan C. Mirro M. (2018). College students’ experiences with, and willingness to use, different types of telemental health resources: do gender, depression/anxiety, or stress levels matter? Telemed. e-Health 24, 998–1005. doi: 10.1089/tmj.2017.0243, PMID: 29658826

[ref59] van Daele T. Karekla M. Kassianos A. P. Compare A. Haddouk L. Salgado J.. (2020). Recommendations for policy and practice of telepsychotherapy and e-mental health in Europe and beyond. J. Psychother. Integr. 30, 160–173. doi: 10.1037/int0000218

[ref60] Venkatesh V. Morris M. G. Davis G. B. Davis F. D. (2003). User acceptance of information technology. Toward a unified view. MIS Q. 27, 425–478. doi: 10.2307/30036540

[ref61] Wang W. Shen F. (2019). The effects of health narratives: Examining the moderating role of persuasive intent. Health Mark. Q. 36, 120–135. doi: 10.1080/07359683.2019.1575061, PMID: 30907268

[ref62] Webelhorst C. Jepsen L. Rummel-Kluge C. (2020). Utilization of e-mental-health and online self-management interventions of patients with mental disorders–A cross-sectional analysis. PLoS One 15:e0231373. doi: 10.1371/journal.pone.0231373, PMID: 32310991 PMC7170258

[ref63] Wopperer J. Apolinário-Hagen J. Wals F. Harrer K. Kemper J. Salewski C.. (2019). Exploring the usefulness of testimonials as a tool to improve the acceptance of e-mental health interventions among university students: preliminary results of a pilot RCT: Poster session. Copenhagen, Denmark.

[ref64] Zebregs S. van den Putte B. Neijens P. Graaf A. (2015). The differential impact of statistical and narrative evidence on beliefs, attitude, and intention: a meta-analysis. Health Commun. 30, 282–289. doi: 10.1080/10410236.2013.842528, PMID: 24836931

