# Supplementary material for: Investigating the Persuasive Effects of Testimonials on the Acceptance of Digital Stress Management Trainings Among University Students and Underlying Mechanisms: A Randomized Controlled Trial
Source: Front Psychol. 2021 Oct 13;12:738950. doi: 10.3389/fpsyg.2021.738950 (PMC8549694; doi:10.3389/fpsyg.2021.738950)
Supplement: Supplementary file 1 [file Data_Sheet_1.pdf]

Supplementary Material 1

Supplementary Material: Methods section – survey and interventions

Stimulus material and questionnaire of the German original and the translated English version of the study material with notes

1 Deutsche Originalversion der Studienmaterialien

(German version of the study material)

Texte und Materialien der Online-Umfrage in Unipark (Auszug)

Vor der Studienteilnahme:

- Studieninformation
- Datenschutzerklärung
- Einverständniserklärung („click to agree“)

<u>Erste Phase: Information und Baseline-Messung</u>

Willkommenstext mit einleitender Information:

Herzlich willkommen bei der Online-Studie zum Einfluss verschiedener Informationsarten auf Akzeptanz und Einstellungen gegenüber professionell begleiteten **Online-Gesundheitstrainings und -therapien**. In dieser Untersuchung wird ein Schwerpunkt auf Angebote im Bereich der mentalen Gesundheit gelegt. Dafür wird hier die Bezeichnung **"E-Mental-Health-Angebote"** gebraucht, abgekürzt mit "**eMH-Angebote**".

Vielen Dank, dass Sie sich die Zeit nehmen, kurze Informationen zu lesen und Fragen zu Ihrer Person und zu Ihren Ansichten über eMH-Angebote zu beantworten. Dafür können Sie im Anschluss die

Bestätigung für eine halbe Versuchspersonenstunde erhalten. Als kleines Dankeschön haben Sie zudem die Möglichkeit, an der Verlosung von drei Buchgutscheinen (jeweils à 20 Euro) teilzunehmen.

Demografische Daten:

Alter (ab 18 Jahren), **Geschlecht** (m/w/d),

höchster Bildungsabschluss (Hauptschulabschluss, Realschulabschluss, Fachhochschulreife, allgemeine Hochschulreife, Meisterbrief, Bachelor, Master/Diplom/Magister, Promotion, Habilitation, Sonstiges – und zwar <Freitext> (anonymisiert)

Status Studierende: eingeschrieben bzw. beurlaubt; **Studienmodell** (Fernstudium, Präsenzstudium, Fern- und Präsenzstudium, Sonstige/Weiterbildungsstudium), **Studienzeit** (Teilzeit, Vollzeit)

Erfahrung mit eMH-Angeboten (ja/nein/weiß nicht), übernommen aus Apolinário-Hagen et al. (2021):

1 Haben Sie schon einmal vor dieser Studie von E-Mental-Health Angeboten gehört?

2 Haben Sie sich schon zu einem (oder mehreren) eMH-Angebot(en) näher informiert?

3 Haben Sie schon ein (oder mehrere) eMH-Angebot(e) genutzt?

Einstellung-prä-Intervention, Kurzabfrage (wahrgenommene Nützlichkeit, basierend u.a. auf UTAUT), zuvor getestet (Apolinário-Hagen et al., 2021)

Im Folgenden geht es um Ihre Erwartungen und persönliche Präferenzen in Bezug auf professionell begleitete eMH-Angebote zum Umgang mit psychischen Problemen bzw. Stress und zur Stärkung von Resilienz (Widerstandsfähigkeit). Persönliche Erfahrungen mit einem konkreten Angebot sind für die Beantwortung der Fragen nicht erforderlich. Bitte wählen sie die Antwortoption, welche am ehesten auf Sie zutrifft. Es gibt keine richtige oder falsche Antwort.

1 Ich finde eMH-Angebote im Allgemeinen hilfreich.

2 Ich finde eMH-Angebote können konventionelle Gesundheitsangebote sinnvoll unterstützen.

3 Ich würde eMH-Angebote im Falle von Stress, psychischen Problemen oder zur Stärkung von Resilienz an Freunde, Bekannte oder Familienmitglieder empfehlen.

(Skalierung: 1-7, Min-Max)

Nutzungsabsicht-Prä-Intervention (Baseline), Kurzabfrage (UTAUT-basiert; adaptiert (Hennemann et al., 2016))

Die folgenden Aussagen beschäftigen sich damit, inwiefern Sie sich vorstellen können, dass Ihnen professionell begleitete eMH-Angebote beim Umgang mit psychischen Problemen bzw. Stress und zur Stärkung von Resilienz (Widerstandsfähigkeit) helfen können. Bitte wählen Sie eine Antwortoption und zwar diejenige, welche am ehesten auf Sie zutrifft. Es gibt keine richtige oder falsche Antwort.

1 Ich kann mir vorstellen, dass eMH-Angebote mein Wohlbefinden fördern können.

2 Ich würde gerne ein eMH-Angebot ausprobieren.

3 Ich plane, in den nächsten Monaten ein eMH-Angebot zu nutzen.

(Skalierung: 1-7)

Wahrgenommener Stress, PSS-10 (Klein et al., 2016)

Die folgenden Aussagen beschäftigen sich damit, wie häufig Sie sich während der letzten zwei Wochen durch Stress belastet fühlten. Bitte wählen Sie eine Antwortoption und zwar diejenige, welche am ehesten auf Sie zutrifft. Es gibt keine richtige oder falsche Antwort.

10 Items (verfügbar über: Klein et al., 2016)

(Skalierung: 1-5, angepasst wegen Uniparkvorgabe von 0-4 auf 1-5)

Zweite Phase: INTERVENTION

Randomisierung, Erklärung für Probanden

Mit Hilfe dieser Studie soll verglichen werden, wie sich unterschiedlich gestaltete Informationstexte auf die Bewertung von eMH-Angeboten auswirken. Sie werden nun einer von vier Gruppen zugeteilt und erhalten verschiedene Informationen zu eMH-Angeboten.

Die Zuteilung erfolgt per Zufallsprinzip, um die Zusammensetzung der Gruppen so zu gestalten, dass gleiche Versuchsbedingungen für alle Teilnehmenden geschaffen werden. Es sollen mögliche Rückschlüsse auf die Rolle verschiedener Informationen zu eMH-Angeboten auf deren Beurteilung durch Studierende untersucht werden. Die dabei erhobenen Daten können dazu beitragen, eMH-Angebote für Studierende ansprechender zu gestalten.

Bitte lesen Sie sich alle dargebotenen Informationen sorgfältig durch. Im Anschluss stellen wir Ihnen Fragen zu Ihrer Meinung über die Angebote.

Einleitungstext: Kontrollgruppe und Interventionsgruppen

Sie erhalten nun Informationen zu eMH-Angeboten. Zur Erinnerung: Unter eMH-Angeboten werden in dieser Untersuchung wissenschaftlich fundierte und professionell begleitete Online-Gesundheitstrainings und -therapien zur Stärkung der psychischen Gesundheit verstanden. Bitte lesen Sie die Texte aufmerksam durch und beantworten Sie im Anschluss Fragen zu Ihrer Meinung dazu. Reine Wissensfragen werden nicht gestellt.

Für Hilfesuchende sollen eMH-Angebote im Bereich der psychischen Gesundheit neue Möglichkeiten zur Verbesserung und Erweiterung des Angebots bieten. Traditionelle Trainings- oder Therapieplätze im Präsenzmodus ("Face-to-Face") sind oft schwer zu erhalten, es bestehen häufig lange Wartelisten. Internetbasierte Angebote werden als eine Möglichkeit angesehen, diese Lücke in der Versorgung zu schließen. Zahlreiche wissenschaftliche Studien belegen die Wirksamkeit von eMH-Angeboten bei einem breiten Spektrum psychischer Probleme. So gibt es zum Beispiel Trainings für den Umgang mit allgemeinem Stressverhalten, mit leichten bis mittelschweren Depressionen, Angst- oder Essstörungen sowie auch zur Stärkung von Resilienz.

Eine mögliche Form von eMH-Angeboten sind internetbasierte begleitete Programme, bei denen Online-Selbstlernmodule bearbeitet werden. Zudem kann online Unterstützung durch eine Therapeutin oder einen Therapeuten erfolgen. Tägliche App-Anwendungen sind möglich. Die Kommunikation mit der Therapeutin bzw. dem Therapeuten erfolgt dabei per E-Mail, Chat oder Videokonferenz.

Es wird nun kurz ein konkretes Beispiel für ein wissenschaftlich geprüftes und professionell therapeutisch begleitetes eMH-Angebot in den Bereichen "Stress" und "Resilienz (Widerstandsfähigkeit)" vorgestellt. Das Training besteht aus sechs aufeinanderfolgenden Online-Einheiten von jeweils ca. einer Stunde Dauer. Die Einheiten können flexibel an verschiedenen Tagen bearbeitet werden. Sie sind textbasiert, enthalten zudem Bilder und kleine Videos. Es können Gedanken notiert, kleinere Aufgaben gelöst und Ziele definiert werden. Eine Therapeutin oder ein Therapeut begleitet den Prozess online. Das Training zielt darauf ab, (wieder) zu mehr Leistungsfähigkeit zu gelangen. Im Stresstraining werden persönliche Themen identifiziert, Bewältigungsstrategien erprobt, Problemlösestrategien eingesetzt und der Umgang mit belastenden Gefühlen gelernt. Im Resilienz-Training werden die Themen Selbstwirksamkeit, Optimismus, Beziehungspflege und Selbstfürsorge behandelt.

Testimonials (Testimonial-Intervention, nur für die Interventionsgruppen)

Aktive Kontrollgruppe: Keine Testimonials

(=> direkt weiter zur Postinterventionsmessung)

Interventionsgruppe 1 (IG1): Testimonials von Berufstätigen (nichtakademischer Arbeitsbereich)

Teilnehmende berichten von ihren Erfahrungen mit dem Training:

- "Den Stress habe ich nach und nach von der Arbeit nach Hause getragen - das führte zu weiteren Spannungen. Das Training hat mir geholfen aus diesem Teufelskreis auszusteigen. Heute geht es mir wieder gut!" (Chris M., Buchhaltung)

- "Es hat mir sehr geholfen!" (Micha T., Gesundheitswesen)

- „Insgesamt kann ich das Training allen empfehlen, die an sich arbeiten wollen und dazu das nötige Rüstzeug brauchen. Mich hat es innerhalb weniger Wochen viel stärker gemacht." (Mo F., Verkauf)

Interventionsgruppe 2 (IG2): Testimonials von Studierenden

Studierende berichten von ihren Erfahrungen mit dem Training:

- "Gut, dass ich dieses Angebot bekommen habe. Meine stressigen Studienphasen habe ich nun im Griff - hat mir super geholfen. Ich fühle mich stark für das, was kommt!" (Mo W., Jura)

- "Ich konnte flexibel in meinem Tempo die Einheiten bearbeiten. So war es möglich, neben dem Studium am Training teilzunehmen. Gut durchdachte Inhalte. Bin daran gewachsen." (Chris S., Psychologie)

- "Vielseitig und interessant. In der Handhabung große Vorteile durch die zeitliche und örtliche Unabhängigkeit. Hat mir viel gebracht - ich empfehle es Kommilitonen und Kommilitoninnen wärmstens!" (Micha K., Sozialwissenschaften)

Interventionsgruppe 3 (IG3): Testimonials von Experten und Expertinnen

Expertinnen und Experten meinen dazu:

- "Das Online-Angebot bietet ein ausgewogenes und gut anwendbares Training, um die Bewältigung von Stress anzugehen. Die Erfahrungen damit sind gut, ich kann es Jedem und Jeder wärmstens empfehlen." (Dr. Klaus Walter, Psychologischer Psychotherapeut)

- „Es werden konkrete Ansätze angeboten, um besser mit Stress umgehen zu können - ihn von vornherein zu vermeiden. Die Möglichkeit individuell wählbarer Zusatzmodule ist hervorzuheben. Damit steigt die Wirksamkeit des Trainings.“ (Prof. Dr. Anne Kanewski, Institut für Psychologie)

- „Besonders anzumerken ist die gute wissenschaftliche Fundierung. Quellen werden genannt, Fachbegriffe und Hintergründe erklärt. Die Ergebnisse sprechen für sich: effektiv und nachhaltig.“ (Prof. Dr. Chris Hurley, Psychiater, Forschungsschwerpunkt Stressbewältigung)

Dritte Phase: Postinterventions-Messung

Glaubwürdigkeit der Quellen, selbstkonstruiert und zuvor getestet (Apolinário-Hagen et al., 2021)

Bitte schätzen Sie eMH-Angebote in Bezug auf die folgenden Aussagen aus Ihrer Sicht ein. Wählen Sie aus, wie stark Sie jeweils zustimmen.

1 Die Angaben fand ich glaubwürdig.

2 Bei den dargebotenen Informationen war in der Regel deutlich zu erkennen, ob es sich um Fakten oder um Meinungen handelte.

3 Die Beschreibungen sind glaubhaft.

(Skalierung: 1-7)

Wahrgenommene Ähnlichkeit (nur für IG1-IG3); selbstkonstruiert und zuvor getestet (Apolinário-Hagen et al., 2021)

Bitte beantworten Sie die folgenden Items, indem Sie sich mit den Personen vergleichen, die ihre Erfahrungen mit dem eMH-Angebot geschildert haben. Lesen Sie jede Aussage und wählen Sie dann aus, wie stark Sie jeweils zustimmen.

1 Der Hintergrund der Personen ist ähnlich wie meiner.

2 Die Personen haben eine ähnliche Bildung wie ich.

3 Die Personen haben ähnliche Belastungen wie ich.

4 Ich fühle mich in ähnlicher Weise betroffen wie die Personen.

5 Die Lebenssituation der Personen ist ähnlich wie meine.

(Skalierung: 1-7)

Einstellung – Post-Intervention, Kurzabfrage (wahrgenommene Nützlichkeit, UTAUT-basiert), zuvor getestet (Apolinário-Hagen et al., 2021)

Sie haben nun einige Informationen zu eMH-Angeboten zum Umgang mit psychischen Problemen bzw. Stress und zur Stärkung von Resilienz erhalten. Im Folgenden geht es nun um Ihre Meinung zu solchen Angeboten. Persönliche Erfahrungen mit einem konkreten Angebot sind für die Beantwortung der Fragen nicht erforderlich. Bitte geben Sie an, was am Ehesten auf Sie zutrifft. Es gibt keine richtige oder falsche Antwort.

1 Solche eMH-Angebote können bei der Bewältigung von (z.B. durch Stress entstandenen) psychischen Problemen helfen.

2 Ich denke, das Nutzen eines eMH-Angebots kann Resilienz (Widerstandsfähigkeit) stärken.

3 Ich würde eMH-Angebote Freunden oder Bekannten, die Stress oder psychische Probleme haben, empfehlen.

(Skalierung: 1-7)

Nutzungsabsicht - Post-Intervention (UTAUT-basiert; adaptiert nach Hennemann et al., 2016)

Inwiefern können Sie sich vorstellen, dass Ihnen eMH-Angebote beim Umgang mit Stress (oder anderen psychischen Problemen) sowie zur Stärkung von Resilienz helfen können? Bitte geben Sie an, was am Ehesten auf Sie zutrifft. Es gibt keine richtige oder falsche Antwort.

1 Ich kann mir vorstellen, dass ein solches Angebot mein Wohlbefinden fördern kann.

2 Ich würde gerne ein solches Angebot ausprobieren.

3 Ich plane ein solches Angebot in der nächsten Zeit zu nutzen.

(Skalierung: 1-7)

Einstellung gegenüber digitalen Gesundheitsinterventionen bzw. Onlinetherapien -Post-Intervention, APOI, und ETAM (Langskalen zur Einstellungsmessung)

Im Folgenden werden Sie zu Ihren Ansichten und Erwartungen zu eMH-Angeboten befragt. Es sind zur Beantwortung keine konkreten Erfahrungen mit Angeboten notwendig. Da es sich um

unterschiedliche, bereits in anderen Untersuchungen zu professionellen Online-Gesundheitstrainings angewendeten Fragebögen handelt, weicht die Wortwahl von den bisherigen Informationstexten ab. Zur besseren Lesbarkeit wurde im ersten Fragebogen die Bezeichnung "Therapeut" gewählt. Sie steht auch für "Therapeutin".

Für den Begriff **"eMH-Angebote"** stehen hier die Bezeichnungen **"psychologische Online-Interventionen"** bzw**. "Internettherapien".**

APOI (Attitude towards Psychological Online Interventions; (Schröder et al., 2015))

Bitte geben Sie an, was am Ehesten auf Sie zutrifft. Es gibt keine richtige oder falsche Antwort.

16 Items (verfügbar über: Schröder et al., 2015)

(Skalierung: 1-5)

ETAM (E-Therapy Attitude Measure; (Apolinário-Hagen et al., 2018))

1 Internettherapien sind modern bzw. entsprechen unserer modernen Zeit.

2 Internettherapien werden konventionelle Psychotherapien zukünftig ersetzen können.

3 Internettherapien lassen sich besser mit Arbeit und Privatleben vereinbaren als konventionelle Psychotherapien.

4 Es macht für mich keinen Unterschied, ob eine Psychotherapie über das Internet oder in der Praxis erfolgt.

5 Internettherapien werden mehr Menschen mit psychischen Problemen erreichen.

6 Krankenkassen sollten die Kosten für Internettherapien übernehmen.

7 Internettherapien sind vergleichbar wirksam wie konventionelle Psychotherapien.

8 Das Vertrauen zu einem Therapeuten/einer Therapeutin kann über das Internet genauso gut aufgebaut werden wie bei konventionellen Psychotherapien.

9 Internettherapien sind eine geeignete Alternative zu konventionellen Psychotherapien.

10 Bei psychischen Problemen würde ich eine Internettherapie in Anspruch nehmen.

11 Ich würde eine Internettherapie einer konventionellen Psychotherapie vorziehen.

12 Internettherapien werden mehr Patienten erreichen und ihnen helfen können.

13 Ich mache mir keine besonderen Sorgen um den Datenschutz bei Internettherapien.

14 Durch die Anonymität bei Internettherapien sinkt die Hemmschwelle, offen und ehrlich über wichtige Probleme zu sprechen.

15 Durch die Verbreitung von Internettherapien werden sich Menschen früher professionelle Hilfe holen.

16 Missverständnisse treten bei Internettherapien ähnlich häufig auf wie bei konventionellen Psychotherapien.

17 Internettherapien eignen sich für die meisten Patienten, unabhängig vom persönlichen Hintergrund (Alter, Geschlecht, Bildung, etc.).

(Skalierung 1-5)

Finale Phase: Abschluss und Debriefing

Seriousness check (=Standarditem der Fakultät für Psychologie für anonyme Online-Befragungen in Verbindung mit dem virtuellen Labor zur Bereitstellung von Versuchspersonenstunden)

In welcher Form haben Sie an der Studie teilgenommen?

- Ich habe ernsthaft teilgenommen und alle Fragen zutreffend beantwortet.
- Ich habe nicht ernsthaft teilgenommen und mich (teilweise) unaufmerksam durchgeklickt und/oder nicht wahrheitsgemäß geantwortet.

Debriefing- Aufklärung zur Studie (nach Studienabschluss), inkl. Informationen zur Begründung der Konstruktion von Testimonials

Einverständnis – Datenverwendung (zweite Abfrage)

Sind Sie damit einverstanden, dass die von Ihnen eingegebenen Daten für den eingangs genannten Forschungszweck und im dort beschriebenen Rahmen genutzt werden? (Ja/Nein)

2 English version of the study material and survey

(translated for publication purposes)

Text-based material of the online survey using Unipark (excerpt=

Prior to study participation:

- Study information
- Data protection declaration / privacy statement
- Informed consent („click to agree“)

First Stage: Information and Baseline-Assessment

Welcome text with introductory information:

Welcome to the online study on the influence of different types of information on the acceptance of and attitudes towards professionally guided **online health training and therapies**. In this study, a focus is placed on offers in the field of mental health. The term "**E-Mental Health Services**" will be used here for this purpose, abbreviated as "**eMH Services**".

Thank you for taking the time to read brief information and answer questions about yourself and your views on eMH services. You can finally receive 0,5 study credits. As a little reward for your participation, you also have the opportunity to participate in the lottery for three book vouchers (each worth 20 euros).

Demographic data:

Age (at least 18 years), **gender** (m / f / d),

Highest educational qualification (secondary school qualification, secondary school qualification, technical college entrance qualification (German “Fachhochschulreife”), general university entrance qualification (German “Allgemeine Hochschulreife” or “Abitur”), master craftsman certificate

(German “Meisterbrief”), bachelor, master / diploma / magister, doctorate, habilitation, other - namely <free text> (anonymized)

Status of students: enrolled or on leave, **study model** (distance learning, on-campus university, distance and on-campus university, other/continuing education program), **study time model** (part-time, full-time)

Experience with eMH services (yes, no, don't know / not sure), adopted from Apolinário-Hagen et al. (2021)

1 Have you ever heard of e-mental health services before this study?

2 Have you already found out more about one (or more) eMH service (s)?

3 Have you already used one (or more) eMH service (s)?

Baseline Attitude, pre-intervention, brief scale (perceived usefulness, based on the UTAUT), pretested (Apolinário-Hagen et al., 2021)

The following part is about your expectations and personal preferences regarding professionally guided eMH services to deal with psychological problems or stress and to strengthen resilience. Personal experience with a specific intervention is not required to answer the questions. Please select the answer option that best applies to you. There is no right or wrong answer.

1 I generally find eMH services helpful.

2 I think eMH services can support conventional health offers in a meaningful way.

3 I would recommend eMH services to friends, acquaintances or family members in the event of stress, psychological problems or to strengthen resilience.

(Scaling: 1-7, min-max)

Baseline Intention to use (pre-intervention), adapted brief scale based on the UTAUT (Hennemann et al., 2016)

The following statements concern the extent to which you can imagine that professionally accompanied eMH services can help you in dealing with psychological problems or stress and in strengthening resilience. Please select an answer option that most closely applies to you. There is no right or wrong answer.

1 I can imagine that eMH services can promote my well-being.

2 I would like to try an eMH service.

3 I plan to use an eMH service in the next few months.

(Scaling: 1-7)

Perceived stress, PSS-10 (German version: Klein et al., 2016)

The following statements deal with the frequency you have felt stressed during the past two weeks. Please select an answer option and the one that most closely applies to you. There is no right or wrong answer.

10 items (available from: Klein et al., 2016)

(Scaling: 1-5, due to Unipark specification, adjusted from 0-4 to 1-5)

Second Stage: INTERVENTION

Randomization, explanation for participants

The aim of this study is to compare how differently designed information texts affect the evaluation of eMH services. You will now be assigned to one of four groups and receive various information about eMH services.

The allocation is made at random in order to structure the composition of the groups in such a way that the same experimental conditions are created for all participants. The goal is to derive possible conclusions about the role of various features of information on eMH services on their assessment by university students. The data collected in the process can help make eMH services more appealing for university students.

Please read all of the information provided carefully. We will then ask you questions about your opinion about the described services.

Introductory text control and intervention groups

You will now receive information on eMH services. As a reminder: In this study, eMH services are understood as scientifically grounded and professionally guided online health trainings and online therapies to strengthen mental health. Please read the texts carefully and then answer questions about your opinion. Pure knowledge questions are not asked.

For those seeking help, eMH services in the field of mental health promotion are intended to offer new opportunities to improve and expand the availability of mental health services. Traditional face-to-face training or therapy options are often difficult to obtain, and there are often long waiting lists.

Internet-based interventions are seen as one way of closing this supply gap. Numerous scientific studies have proven the effectiveness of eMH services for a wide range of psychological problems. For example, there are training courses for dealing with stress, mild to moderate depression, anxiety or eating disorders, and also for strengthening resilience.

One possible type of eMH services are internet-based guided programs in which online self-learn modules are offered. In addition, online support can be provided by a therapist. Daily app-based reminders can be included. Communication with the therapist takes place via email, chat or video conference software.

A concrete example of a scientifically tested and professionally guided, therapist-supported eMH service in the areas of "stress" and "resilience" will now briefly be presented. The training consists of six consecutive online units of about one hour each. The units can be processed flexibly on different days. They are text-based and also contain pictures and brief videos. Thoughts can be documented, brief tasks can be solved and goals can be defined. A therapist accompanies the process online. The aim of the training is to achieve greater performance (again). In the stress training, personal problems are identified, coping strategies are tested, problem-solving strategies are used and how to deal with stressful feelings is learned. In the resilience training, the topics of self-efficacy, optimism, maintaining relationships and self-care are dealt with.

Testimonials (testimonial intervention, IGs only)

Active control group (aCG): no testimonials

(=> directly redirected to the post-intervention assessment)

Intervention group 1 (IG1): testimonials from staff (non-academic work area)

Participants report on their experiences with the training:

- "I gradually carried the stress home from work - that led to further tension. The training helped me to get out of this vicious circle. Today I'm fine again!" (Chris M., Accounting)
- "It helped me a lot!" (Micha T., Healthcare)
- “Overall, I can recommend the training to everyone who wants to work on themselves and needs the necessary tools. It made me a lot stronger within a few weeks." (Mo F., Sales)

Intervention group 2 (IG2): testimonials from university students

University students report on their experiences with the training:

- "It's good that I received this offer. I now have my stressful study phases under control - it helped me a lot. I feel strong for what's coming!" (Mo W., Jurisprudence)
- "I was able to work on the units flexibly at my own pace. This made it possible to take part in training alongside my studies. Well thought-out content. I grew from it." (Chris S., Psychology)
- "Versatile and interesting. Great advantages in handling due to the independence of time and location. It helped me a lot - I warmly recommend it to fellow university students!" (Micha K., Social Sciences)

Intervention group 3 (IG3): Testimonials from experts

Experts say about the training:

- “The online service offers a balanced and easily applicable training for stress coping. The experience with it has been good, I can warmly recommend it to everyone." (Dr. Klaus Walter, psychological psychotherapist)
- “Concrete approaches are offered to be able to better deal with stress – or to prevent it in advance. The possibility of individually selectable bonus modules should be emphasized. This increases the effectiveness of the training." (Prof. Dr. Anne Kanewski, Institute for Psychology)
- “Particularly noteworthy is the good scientific foundation. Sources are named, technical terms and backgrounds are well explained. The results speak for themselves: effective and sustainable." (Prof. Dr. Chris Hurley, psychiatrist, research focus on stress management)

Third stage: Post-intervention Assessment

Source credibility; self-constructed and pretested (Apolinário-Hagen et al., 2021)

Please assess eMH services in relation to the following statements from your point of view. Choose how much you agree with each.

1 I found the information credible.

2 As a rule, it was clear from the information presented whether it was facts or opinions.

3 The descriptions are credible.

(Scaling: 1-7)

Perceived similarity (only presented to IG1-IG3); self-constructed and pretested (Apolinário-Hagen et al., 2021)

Please answer the following questions by comparing yourself with the people who have described their experiences with the eMH offer. Please read each statement, then choose how much you agree with each one.

1 The background of the people is similar to mine.

2 The people have a similar education to me.

3 The people have stress levels similar to mine.

4 I feel affected in a similar way to the people.

5 The life situation of the people is similar to mine.

(Scaling: 1-7)

Attitude – post-intervention, short scale (perceived usefulness; UTAUT-based), pretested (Apolinário-Hagen et al., 2021)

You have now received some information on eMH services for dealing with psychological problems or stress and for strengthening resilience. The following is your opinion on such offers. Personal experience with a specific offer is not required to answer the questions. Please indicate what is most likely to apply to you. There is no right or wrong answer.

1 Such eMH offers can help to cope with psychological problems (e.g. caused by stress).

2 I think using an eMH service can strengthen resilience.

3 I would recommend eMH offers to friends or acquaintances who have stress or psychological problems.

(Scaling: 1-7)

Intention to use, post-intervention, short scale (UTAUT-based; adapted from Hennemann et al. 2016)

To what extent can you imagine that eMH services can help you in dealing with stress (or other psychological problems) and in strengthening resilience? Please indicate what is most likely to apply to you. There is no right or wrong answer.

1 I can imagine that such an offer can promote my well-being.

2 I would like to try out such an offer.

3 I plan to use such an offer in the near future.

(Scaling: 1-7)

Attitudes towards digital interventions / online therapies –post-intervention, APOI and ETAM (attitude full scales)

In the following, you will be asked about your views and expectations about eMH services. No specific experience with such offers is necessary to answer the questions. Since we use different questionnaires that have already been applied in other studies on professional online health trainings, the choice of words differs from the previous information texts. Specifically, the terms "psychological online interventions" or "internet therapies" stand for the term "eMH services".

APOI (attitude towards psychological online interventions; Schröder et al., 2015)

Please indicate what is most likely to apply to you. There is no right or wrong answer.

16 items (available from: Schröder et al., 2015)

(Scaling: 1-5)

ETAM (e-therapy attitude measure; Apolinário-Hagen et al., 2018)

1 Internet-based therapies are modern and in line with our modern times.

2 Internet-based therapies will replace conventional face-to-face psychotherapy in the future.

3 Internet-based therapy is more compatible with work and private life than conventional face-to-face therapy.

4 It makes no difference to me whether psychotherapy is conducted through the internet or in a psychotherapy practice in a clinic.

5 Internet-based therapies will reach more individuals with mental health problems.

6 Health insurance companies should cover the costs for internet-based therapies.

7 Internet-based therapy programs are as effective as conventional face-to-face psychotherapies.

8 Trust in a therapist can be just as easily built on the internet as in conventional face-to-face psychotherapy.

9 Internet-based therapies are an appropriate alternative to conventional face-to-face psychotherapy.

10 In case of mental health problems, I would attend an internet-based therapy.

11 I would prefer an internet-based therapy to a conventional face-to-face psychotherapy.

12 Internet-based therapies will reach more patients and help them.

13 I'm not particularly worried about data security in internet therapies.

14 The anonymity in internet therapies decreases the threshold to speak openly and honestly about important issues.

15 Through the dissemination of internet therapies, persons will get professional help earlier.

16 Misunderstandings occur in internet therapies as often as in conventional psychotherapies.

17 Internet therapies are suitable for most patients, regardless of their personal background (age, sex, education, etc.).

(scaling: 1-5)

Final stage: Ending and debriefing

Seriousness check (=> standard item of the Faculty of Psychology for anonymous online surveys in connection with the virtual laboratory to provide course credits)

In what form did you take part in the study?

- I took part seriously and answered all questions correctly.
- I did not take part seriously and clicked my way through (partially) inattentively and / or did not answer truthfully.

Debriefing information on the study (after study completion), including information on the rationale behind the construction of testimonials

Consent – data use (second request)

Do you agree that the data you have entered may be used for the research purpose mentioned above and in the context described there? (Yes, No)

****

Abbreviations

aCG: active control group (information only)

APOI: attitudes towards psychological online interventions (questionnaire)

eMHS: electronic mental health service/s

ETAM: e-therapy attitude measure

IG: intervention group (receiving information plus testimonials)

PSS-10: perceived stress scale, 10 items

UTAUT: unified theory of acceptance and use of technology

3 References

Apolinário-Hagen, J., Harrer, M., Dederichs, M., Fritsche, L., Wopperer, J., Wals, F., Loerbroks, A. et al. (2021). Exploring the influence of testimonial source on attitudes towards e-mental health interventions among university students: Four-group randomized controlled trial. *PloS one* 16, e0252012. doi: 10.1371/journal.pone.0252012.

Apolinário-Hagen, J., Harrer, M., Kählke, F., Fritsche, L., Salewski, C., and Ebert, D. D. (2018). Public Attitudes Toward Guided Internet-Based Therapies: Web-Based Survey Study. *JMIR Ment Health* 5, e10735. doi: 10.2196/10735.

Hennemann, S., Beutel, M. E., and Zwerenz, R. (2016). Drivers and Barriers to Acceptance of Web-Based Aftercare of Patients in Inpatient Routine Care. A Cross-Sectional Survey. *J Med Internet Res* 18, e337. doi: 10.2196/jmir.6003.

Klein, E. M., Brähler, E., Dreier, M., Reinecke, L., Müller, K. W., Schmutzer, G., Wölfling, K., and Beutel, M. E. (2016). The German version of the Perceived Stress Scale – psychometric characteristics in a representative German community sample. *BMC psychiatry* 16. doi: 10.1186/s12888-016-0875-9.

Schröder, J., Sautier, L., Kriston, L., Berger, T., Meyer, B., Späth, C., Köther, U., Nestoriuc, Y., Klein, J. P., and Moritz, S. (2015). Development of a questionnaire measuring Attitudes towards Psychological Online Interventions-the APOI. *Journal of affective disorders* 187, 136–141. doi: 10.1016/j.jad.2015.08.044.
