# Supplementary material for: Investigating the Persuasive Effects of Testimonials on the Acceptance of Digital Stress Management Trainings Among University Students and Underlying Mechanisms: A Randomized Controlled Trial
Source: Front Psychol. 2021 Oct 13;12:738950. doi: 10.3389/fpsyg.2021.738950 (PMC8549694; doi:10.3389/fpsyg.2021.738950)
Supplement: Supplementary file 2 [file Data_Sheet_2.pdf]

Supplementary Material 2

Supplementary Tables and Figures: Results section –ancillary analyses

1 Supplementary material: perceived stress at baseline

Table S1. *Descriptive data on perceived stress in the study arms.*

Study arm	*Mean*	*SD*	*n*
aCG (information only)	3.14	0.42	55
IG1 (added staff testimonials)	3.21	0.34	60
IG2 (added students´ testimonials	3.17	0.34	58
IG3 (added experts´ testimonials)	3.21	0.37	58
**Total**	3.18	0.37	231

Notes. Perceived stress was measured at baseline using the Perceived Stress Scale (PSS-10). Abbreviations: aCG=active control group ("information only"), IG=narrative intervention group (IG), IG1=information plus testimonials by nonacademic staff (i.e., unspecified occupation, work area outside of academia, such as sale), IG2=information plus testimonials by university students, IG3=information plus testimonials by experts. Scale range (min-max): 1-5.

Regarding perceived stress, as measured using the PSS-10, a one-way ANOVA demonstrated no significant differences between the four study arms at the baseline assessment, $F_{(3,227)}$=0.41, p=.747, η_p^2=.005. Post hoc tests were thus not performed. Correlations between perceived stress and other variables are shown in Table S11.

2 Supplementary material: Study flow, randomization and data handling

Figure S1. *Flow Chart on participant flow and data handling.*

(Adapted) CONSORT 2010 Flow Diagram – Data Handling

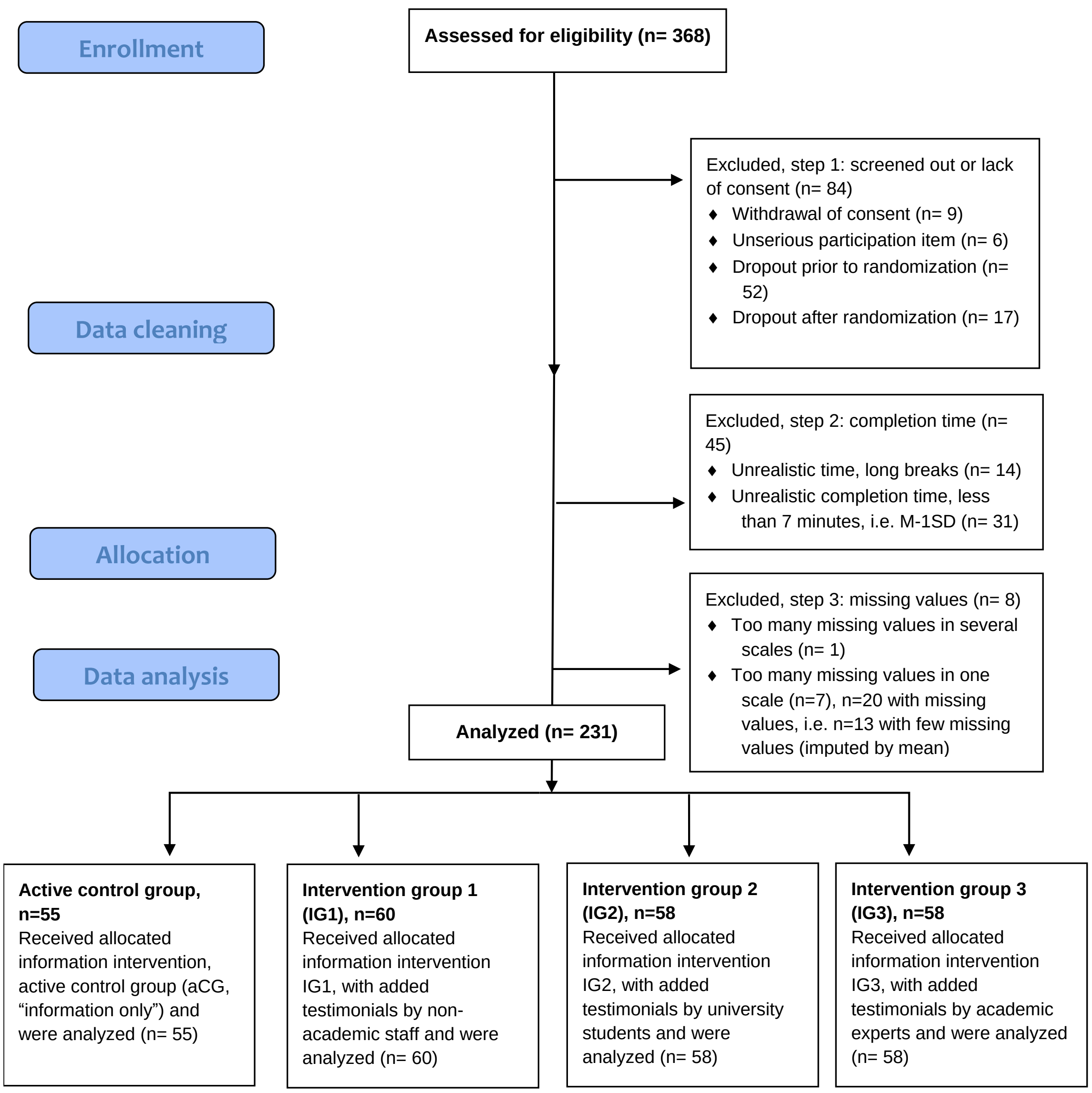

Notes. CONSORT reference: Moher D, Hopewell S, Schulz KF, Montori V, Gøtzsche PC, Devereaux PJ, Elbourne D, Egger M, Altman DG. CONSORT 2010 explanation and elaboration: updated guidelines for reporting parallel group randomised trials. J Clin Epi 2010; 63(8): e1-e37.

3 Supplementary material: Source credibility – ancillary analysis at the post-intervention assessment

Table S2. *Descriptive data on source credibility in the study arms.*

Study arm	*Mean*	*SD*	*n*
aCG (information only)	5.50	1.03	55
IG1 (added staff testimonials)	5.27	0.98	60
IG2 (added students´ testimonials	5.28	0.82	58
IG3 (added experts´ testimonials)	4.94	0.94	58
**Total**	5.25	0.96	231

Notes. The mediator source credibility was measured at the post-intervention assessment. Abbreviations: aCG=active control group (”information only”), IG=narrative intervention group (IG), IG1=information plus testimonials by non-academic staff, IG2=information plus testimonials by university students, IG3=information plus testimonials by experts. Scale range (min-max): 1-7.

Regarding source credibility, a one-way ANOVA demonstrated statistically significant differences between the study arms, $F_{(3,227)}$=3.34, p=.020, η_p^2=.042 (small effect size).

Post hoc tests are shown in Table S3. Difference in source credibility ratings were only found in the comparison of the aCG with IG3. Participants in the aCG (“information only”) found the provided information more credible than participants in the IG3 who received information plus experts´ testimonials (p=.012).

Table S3. *Differences between the experimental groups in source credibility of information on digital mental health interventions (multiple comparisons).*

					**95% CI**	
**(I)**	**(J)**	**Δ *M* (I-J)**	***SD***	***p***	***LL***	***UL***
aCG	IG1	0.225	0.176	1.00	-0.244	0.694
	IG2	0.215	0.178	1.00	-0.257	0.688
	IG3	**0.554***	**0.178**	**.012**	**0.082**	**1.027**
IG1 **(staff)**	aCG	-0.225	0.176	1.00	-0.694	0.244
	IG2	-0.009	0.174	1.00	-0.472	0.453
	IG3	0.330	0.174	.354	-0.133	0.792
IG2 **(student)**	aCG	-0.215	0.178	1.00	-0.688	0.257
	IG1	0.009	0.174	1.00	-0.453	0.472
	IG3	0.339	0.175	.325	-0.127	0.805
IG3 **(expert)**	aCG	**-0.554***	**0.178**	**.012**	**-1.027**	**-0.082**
	IG1	-0.330	0.174	.354	-0.792	0.133
	IG2	-0.339	0.175	.325	-0.805	0.127

Notes. N=231. Post hoc tests with Bonferroni adjustment, dependent variable: source credibility of information on digi-MHSs for stress management purposes, independent variable: study arm (information intervention). Δ *M* = Mean difference, *SD* = standard deviation, 95% CI = 95% confidence interval; *LL* = lower limits, *UL* = upper limits, *p < .05, **p < .01, ***p < .001. Abbreviations (study arms): aCG=active control group (”information only”), IG=narrative intervention group (IG), IG1=information plus testimonials by nonacademic staff, IG2=information plus testimonials by university students, IG3=information plus testimonials by experts.

4 Supplementary material: Source credibility– ancillary analysis at the post-intervention assessment

Table S4. *Descriptive data on perceived similarity in the intervention groups.*

Study arm	*Mean*	*SD*	*n*
IG1 (added staff testimonials)	3.96	1.35	60
IG2 (added students´ testimonials	4.89	0.99	58
IG3 (added experts´ testimonials)	3.79	0.88	58
**Total**	4.21	1.19	176

Notes. The mediator perceived similarity was measured at post-intervention. Abbreviations: IG=narrative intervention group (IG), IG1=information plus testimonials by nonacademic staff, IG2=information plus testimonials by university students, IG3=information plus testimonials by experts. Scale range (min-max): 1-7.

One-way ANOVA demonstrated differences between the intervention groups (IGs) in perceived similarity, $F_{(2,173)}$=16.88, p<.001, η_p^2=.163 (large effect size). As shown in Table S5, perceived similarity was higher in IG2 (students´ testimonials) compared to IG1 (staff) and IG3 (experts).

Table S5. *Differences between the intervention groups in perceived similarity with testimonial sources regarding information on digital mental health interventions (multiple comparisons).*

					**95% CI**	
**(I)**	**(J)**	**Δ *M* (I-J)**	***SD***	***p***	***LL***	***UL***
IG1	IG2	**-0.933***	**0.202**	**<.001**	**-1.422**	**-0.444**
**(staff)**	IG3	0.164	0.202	1.00	-0.325	0.653
IG2	IG1	**0.933***	**0.202**	**<.001**	**0.444**	**1.422**
**(student)**	IG3	**1.097***	**0.204**	**<.001**	**0.603**	**1.590**
IG3	IG1	-0.164	0.202	1.00	-0.653	0.325
**(expert)**	IG2	**-1.097***	**0.204**	**<.001**	**-1.590**	**-0.603**

Notes. N=231. Post hoc tests with Bonferroni adjustment; dependent variable: perceived similarity with testimonial sources regarding digi-MHSs for stress management purposes, independent variable: intervention group (IG; testimonial intervention: IG1, IG2 or IG3). Δ *M* = Mean difference, *SD* = Standard deviation, 95% CI = 95% confidence interval; *LL* = lower limits, *UL* = upper limits, *p < .05, **p < .01, ***p < .001. Abbreviations (study arms): IG=narrative intervention group (IG), IG1=information plus testimonials by staff, IG2=information plus testimonials by university students, IG3=information plus testimonials by experts.

5 Supplementary material: Testimonial effects - ancillary analyses on attitudes and intentions (Research questions 1 and 2)

Preliminary analyses

At baseline, no difference in intentions ($t_{(229)}$=-1.55, p=.124) and attitudes ($t_{(229)}$=-1.83, p=.069) regarding digital mental health services (digi-MHSs), as measured using the short scales, were found between the aCG (n=55) and the three IGs (n=176, see RQ1; independent samples t-Test).

In addition, according to one-way ANOVA, no baseline differences were identified between the four study arms in and attitudes ($F_{(3, 227)}$=1.23, p=.301) and intentions ($F_{(3, 227)}$=2.41, p=.068; see RQ2).

In view of insignificant differences and the brief period between the pre-post-design assessments, we analyzed post-intervention scores of main outcomes in RQ1 and RQ2 (one-way ANOVA) and controlled for the influence baseline values as covariates in RQ2 (one-way ANCOVA; see, Results section in the manuscript on sensitivity analyses).

Attitudes towards digi-MHSs – Research question 2

Table S6. *Descriptive data on attitudes towards digital mental health services in the study arms (without inclusion of baseline values as covariate).*

Study arm	*Mean*	*SD*	*n*
aCG (information only)	5.16	0.99	55
IG1 (added staff testimonials)	5.52	1.01	60
IG2 (added students´ testimonials	5.53	0.78	58
IG3 (added experts´ testimonials)	5.30	0.78	58
**Total**	5.38	0.90	231

Notes. Dependent variable: attitude towards digi-MHSs at post-intervention (short scale); independent variable: experimental group (study arm). Abbreviations: aCG=active control group (”information only”), IG=narrative intervention group (IG), IG1=information plus testimonials by staff, IG2=information plus testimonials by university students, IG3=information plus testimonials by experts. Scale range (min-max): 1-7.

Table S7. *Estimated marginal means for post-intervention attitudes towards digital mental health services in the study arms after the adjustment with baseline values as covariate.*

Study arm	*Mean* [a]	*SE*	95% CI	
			LL	*UL*
aCG	5.26	.104	5.06	5.47
IG1	5.48	.099	5.28	5.67
IG2	5.48	.101	5.28	5.68
IG3	5.30	.101	5.10	5.50

Notes. N=231.Mean[a]: a=Covariates appearing in the model were evaluated based on the value of baseline attitudes (=5.23). Dependent variable: attitude towards digi-MHSs at post-intervention (short scale, perceived usefulness); covariate: baseline attitude; independent variable: experimental group (study arm). Abbreviations: aCG=active control group (”information only”), IG=narrative intervention group (IG), IG1=information plus testimonials by staff, IG2=information plus testimonials by university students, IG3=information plus testimonials by experts; SE=standard error; , 95% CI = 95% confidence interval; *LL* = lower limits, *UL* = upper limits. Scale range (min-max): 1-7.

After adjusting for the statistical influence of baseline attitudes as covariate, post-intervention attitudes, as measured using the short scale, did not differ significantly between the four information groups, $F_{(3, 226)}$=1.27, p=.285, η_p^2=.017, R^2=.29, adjusted R^2=.28 (small effect size).

Intentions to use digi-MHSs – Research question 2

Table S8. *Descriptive data on intentions to use digital mental health services in the study arms (without inclusion of baseline values as covariate).*

Study arm	*Mean*	*SD*	*n*
aCG (information only)	4.29	1.15	55
IG1 (added staff testimonials)	4.83	1.09	60
IG2 (added students´ testimonials	5.35	1.08	58
IG3 (added experts´ testimonials)	4.40	0.98	58
**Total**	4.72	1.149	231

Notes. Dependent variable: intentions to use digi-MHSs at post-intervention; independent variable: experimental group (study arm). Abbreviations: aCG=active control group (”information only”), IG=narrative intervention

group (IG), IG1=information plus testimonials by staff, IG2=information plus testimonials by university students, IG3=information plus testimonials by experts. Scale range (min-max): 1-7.

Table S9. Estimated marginal means for post-intervention intentions to use digital mental health services in the study arms after the adjustment with baseline values as covariate.

			95% CI	
**Study arm**	***Mean*** [a]	***SE***	***LL***	***UL***
aCG	4.45	.089	4.27	4.62
IG1	4.59	.086	4.42	4.76
IG2	5.35	.086	5.18	5.52
IG3	4.49	.087	4.32	4.67

Notes. N=231. Mean[a]: a=Covariates in the model were evaluated based on the value of baseline intentions (=4.53). Dependent variable: intentions to use digi-MHSs at post-intervention; covariate: baseline intentions; independent variable: experimental group (study arm). Abbreviations: aCG=active control group (”information only”), IG=narrative intervention group (IG), IG1=information plus testimonials by staff, IG2=information plus testimonials by university students, IG3=information plus testimonials by experts; SE=standard error; 95% CI = 95% confidence interval; *LL* = lower limits, *UL* = upper limits. Scale range (min-max): 1-7.

After adjusting for baseline intentions, post-intervention intentions differed statistically significant between the information groups, $F_{(3, 226)}$=23.92, p<.001, η_p^2=.24, R^2=.68, adjusted R^2=.67 (large effect size), as shown in the manuscript in Table 2.

In contrast to Table 2 showing that IG2 (students´ testimonials) differed significantly from the other three groups after including the covariate (one-way ANCOVA), the post hoc tests of the one-way ANOVA (without covariate adjustment) indicated no significant difference with IG1 (staff testimonials, p=.054). As shown in Table S10, Bonferroni-adjusted post hoc tests showed higher intentions to use digi-MHSs after exposure to testimonials by students compared to expert testimonials (*Mean* difference=0.95, 95% CI [0.422, 1.486]), p<.001).

Table S10. *Differences between the experimental groups in intentions to use digital mental health interventions (multiple comparisons).*

					95% CI	
**(I)**	**(J)**	**Δ *M* (I-J)**	***SD***	***p***	***LL***	***UL***
aCG	IG1	-0.537*	0.201	.049	-1.072	-0.002 +
	IG2	-1.060***	0.203	<.001	-1.599	-0.520 +
	IG3	-.1060	0.203	1.000	-0.645	0.434
IG1 **(staff)**	aCG	0.537*	0.201	.049	0.002	1.072 +
	IG2	-0.523	0.198	.054	-1.051	0.005
	IG3	0.431	0.198	.184	-0.097	0.959
IG2 **(student)**	aCG	1.060***	0.203	<.001	0.520	1.599 +
	IG1	0.523	0.198	.054	-0.005	1.051
	IG3	0.954***	0.200	<.001	0.422	1.486 +
IG3 **(expert)**	aCG	0.106	0.203	1.000	-0.434	0.645
	IG1	-0.431	0.198	.184	-0.959	0.097
	IG2	-0.954***	0.200	<.001	-1.486	-0.422 +

Notes. N=231. Post hoc tests with Bonferroni adjustment, dependent variable: intentions to use digi-MHSs for stress management purposes, independent variable: study group (information intervention). Δ *M* = Mean difference, *SD* = Standard deviation, 95% CI = 95% confidence interval; *LL* = lower limits, *UL* = upper limits, *p < .05, **p < .01, ***p < .001 + CI means significant if the confidence interval does not include the number zero (significant). Abbreviations (study arms): aCG=active control group (”information only”), IG=narrative intervention group (IG), IG1=information plus testimonials by nonacademic staff, IG2=information plus testimonials by university students, IG3=information plus testimonials by experts.

Table S11. *Correlation between the assessed variables.*

Scale		Attitude baseline	Intention baseline	PSS stress	Similarity post	Credibility post	Attitude post	Intention post	APOI attutude	ETAM attitude
**Attitude – PU / baseline**	*r*	**1.00**	**.557****	**.036**	**.200****	**.289****	**.526****	**.482****	**.452****	**.400****
	p		<.001	.584	.008	<.001	<.001	<.001	<.001	<.001
	n	231	231	231	176	231	231	231	231	231
**Intention - baseline**	*r*	**.557****	**1.00**	**.206****	**.245****	**.164***	**,452****	**.758****	**.458****	**.388****
	p	<.001		.002	.001	.013	<.001	<.001	<.001	<.001
	n	231	231	231	176	231	231	231	231	231
**PSS-10 - Stress / baseline**	*r*	**.036**	**.206****	**1.00**	**.280****	**.058**	**.053**	**.159***	**.010**	**.161***
	p	.584	.002		<.001	.380	.427	.015	.874	.014
	n	231	231	231	176	231	231	231	231	231
**Similarity - post**	*r*	**.200****	**.245****	**.280****	**1.00**	**.194****	**.251****	**.452****	**.134**	**.108**
	p	.008	.001	<.001		.010	.001	<.001	.076	.154
	n	176	176	176	176	176	176	176	176	176
**Credibility - post**	*r*	**.289****	**.164***	**.058**	**.194****	**1.00**	**.400****	**.203****	**.312****	**.281****
	p	<.001	.013	.380	.010		<.001	.002	<.001	<.001
	n	231	231	231	176	231	231	231	231	231
**Attitude – PU / post**	*r*	**.526****	**.452****	**.053**	**.251****	**.400****	**1.00**	**.598****	**.583****	**.508****
	p	<.001	<.001	.427	.001	<.001		<.001	<.001	<.001
	n	231	231	231	176	231	231	231	231	231
**Intention - post**	*r*	**.482****	**.758****	**.159***	**.452****	**.203****	**.598****	**1.00**	**.482****	**.445****
	p	<.001	<.001	.015	<.001	.002	<.001		<.001	<.001
	n	231	231	231	176	231	231	231	231	231
**APOI – Attitude - post**	*r*	**.452****	**.458****	**.010**	**.134**	**.312****	**.583****	**.482****	**1.00**	**.764****
	p	<.001	<.001	.874	.076	<.001	<.001	<.001		<.001
	n	231	231	231	176	231	231	231	231	231
**ETAM – Attitude / post**	*r*	**.400****	**.388****	**.161***	**.108**	**.281****	**.508****	**.445****	**.764****	**1.00**
	p	<.001	<.001	.014	.154	<.001	<.001	<.001	<.001	
	n	231	231	231	176	231	231	231	231	231

Notes. N=231. Perceived similarity was only assessed in the three narrative intervention groups (n=176). Correlation coefficient *r* (Pearson´s *r*), *p* (p-value, 2-fold). * $p < .05$, ** $p < .01$; Abbreviations: APOI=Attitudes towards Psychological Online Interventions (attitudes towards online interventions such as online therapies by clients; clinical context); ETAM=E-Therapy Measure (public attitudes towards online psychotherapy); PSS-10=Perceived Stress Scale with 10 items; PU=perceived usefulness; post=post-intervention assessment.
